# Down-regulation of microRNA-125b-2-3p is a risk factor for a poor prognosis in hepatocellular carcinoma

**DOI:** 10.1080/21655979.2021.1921549

**Published:** 2021-05-05

**Authors:** He-Qing Huang, Gang Chen, Dan-Dan Xiong, Ze-Feng Lai, Li-Min Liu, Ye-Ying Fang, Jin-Hai Shen, Xiang-Yu Gan, Liu-Feng Liao, Yi-Wu Dang

**Affiliations:** a Department of Pathology, The First Affiliated Hospital of Guangxi Medical University, Nanning, Guangxi Zhuang Autonomous Region, P. R. China; b Center for Pharmaceutical Research, Pharmaceutical College, Guangxi Medical University, Nanning, Guangxi Zhuang Autonomous Region, P. R. China; c Department of Radiotherapy, The First Affiliated Hospital of Guangxi Medical University, Nanning, Guangxi Zhuang Autonomous Region, P. R. China; d Department of Pharmacy, Guangxi Medical University Cancer Hospital, Nanning, Guangxi Zhuang Autonomous Region, P.R. China; e Department of Drug Toxicology, Pharmaceutical College, Guangxi Medical University, Nanning, Guangxi Zhuang Autonomous Region, P. R. China

**Keywords:** miR-125b-3p, hepatocellular carcinoma, prognosis, RT-qPCR, nitidine chloride, standard mean difference

## Abstract

Hepatocellular carcinoma (HCC) is a leading cause of mortality in cancer patients, but the association between miR-125b-2-3p and the onset and prognosis of HCC has not been reported in previous studies; thus, the clinicopathological implications of miR-125b-2-3p in HCC require elaboration. To examine the expression of miR-125b-2-3p in HCC, both in-house RT-qPCR and public datasets were used to calculate the standard mean difference (SMD) and the summary receiver operating characteristic (sROC). MiR-125b-2-3p was markedly lower in HCC than in non-tumor tissue as assessed by the in-house RT-qPCR which was confirmed by the integrative analysis showing the SMD being −0.69 and the area under the curve (AUC) being 0.84 based on 1,233 cases of HCC and 630 cases of non-HCC controls. To gain a overview of the clinical value of miR-125b-2-3p in HCC, all possible datasets were integrated, and lower miR-125b-2-3p levels could lead to poorer differentiation and a more advanced clinical stage of HCC. The hazard ratio (HR) of miR-125b-2-3p was also calculated using a Cox proportional hazards model, and the miR-125b-2-3p level could act as an protective indication for the survival with the HR being 0.74 based on 586 cases of HCC. Furthermore, the effect of nitidine chloride (NC), a natural bioactive phytochemical alkaloid, on the regulation of miR-125b-2-3p and its potential targets was also investigated. The miR-125b-2-3p level was increased after NC treatment, while the expression of its potential target PRKCA was reduced. Above all, a low-expressed level of miR-125b-2-3p plays a tumor suppressive role in HCC.

## GRAPHICAL ABSTRACT

**Figure:**
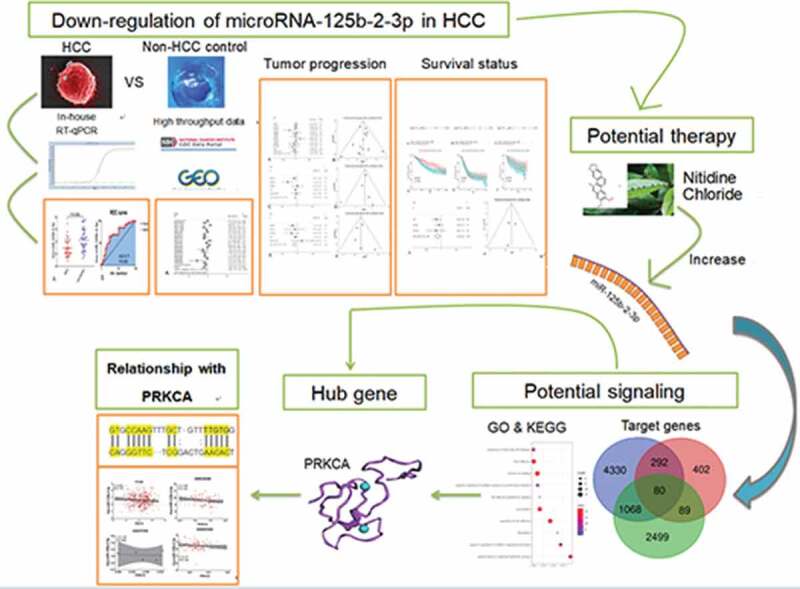

## 1. Introduction

Liver cancer is a leading cause of mortality in patients who present with solid tumors, and the prognosis in advanced patients is poor [1–6]. In the United States, intrahepatic bile duct cancer and liver cancer lead as the fifth-highest mortality rate in males and the seventh highest in females, respectively, and the two tumors pose serious risks to people’s health[7]. In China, liver cancer is the third-highest cause of cancer-related death[8]. Primary liver carcinoma consists mainly of hepatocellular carcinoma (HCC) in liver cells and intrahepatic cholangiocarcinoma (ICC) in biliary epithelial cells. The basic risk factors that are currently recognized for HCC and ICC are cirrhosis, viral hepatitis, aflatoxin and environmental effects [3,9–12]. A series of biological characteristics, such as quick progression, early invasion, and metastasis, is often seen in HCC patients [13–16]. However, the pathogenesis of HCC is not well-defined; in particular, the molecular mechanism remains unclear. Therefore, the molecular mechanism and potential targets of HCC still need to be explored.

MicroRNA (miRNA) are short, endogenous, single-strand RNA molecules that regulate gene expression. They specifically inhibit protein translation and mRNA transcription by interacting with the 3′-untranslated region (3′-UTR) of their targets [17–21]. Many studies have confirmed that deregulation of miRNA is associated with the onset and development of malignant tumors [22–27]. miR-125b-2-3p, also called hsa-miR-125b-2#, is a type of miRNA that has been verified as only slightly expressed in colon cancer and HCC, and it is considered a protective factor in the above two cancers [28,29]. However, the only previous study of miR-125b-2-3p in HCC included data from only a small number of cases, with 12 pairs of HCC and non-HCC liver tissue[29]. In addition, the association between miR-125b-2-3p and the onset and prognosis of HCC was not reported in that study. Therefore, the function of miR-125b-2-3p in HCC remains unknown.

Hence, a summary analysis that combined an in-house quantitative reverse transcription polymerase chain reaction (RT-qPCR) and high throughput data was carried out in our study. We included 1,233 HCC samples and 630 non-tumor samples to thoroughly investigate the relationship between miR-125b-2-3p and HCC. In addition, the clinical significance and the molecular biological functions of miR-125b-2-3p were assessed.

Hence, we hypothesized that miR-125b-2-3p might play a part in the occurrence and development of HCC, and we also required to explore whether miR-125b-2-3p might have potential in the treatment of HCC in the future. A comprehensive analysis was carried out to study the clinical significance of miR-125b-2-3p in HCC, which combined data from in-house quantitative reverse transcription polymerase chain reaction (RT-qPCR) and high throughput data mining. In vivo experiments were used to explore the therapeutic value of miR-125b-2-3p in HCC as influenced by Nitidine Chloride, an extract of traditional Chinese Medicine.

## 2. Materials and methods

### 2.1 Differential expression of miR-125b-2-3p

#### 2.1.1 The miR-125b-2-3p expression value in hepatocellular carcinoma tissue detected by in-house RT-qPCR

To gauge the clinicopathological implications of miR-125b-2-3p in HCC, 26 pairs of HCC formalin-fixed paraffin-embedded tissue and their corresponding non-tumorous liver tissue were acquired from the Department of Pathology at the First Affiliated Hospital of Guangxi Medical University (Nanning, Guangxi, People’s Republic of China). The HCC and non-tumorous liver tissue samples were randomly obtained from surgically resected patients. All experimental protocols were authorized by the Institutional Ethics Committee (IEC) of the First Affiliated Hospital of Guangxi Medical University, and both the clinical doctors and the patients provided informed consent for the samples to be used in this research. The total amount of RNA was isolated and then subjected to complementary DNA (cDNA) synthesis using the TaqMan Reagent, as recommended in the manufacturer’s instructions. Next, 20 µl of cDNA were reversed along with 1 µg RNA, and RT-qPCR was carried out using a miScript SYBR‑Green PCR kit with the 7900HT PCR system (Applied Biosystems; Thermo Fisher Scientific, Inc., Waltham, MA, US). The primer was provided by Applied Biosystems (Thermo Fisher Scientific, Inc.), and the miR-125b-2-3p sequence was UCACAAGUCAGGCUCUUGGGAC (cat. no. 4427975‑002158). The miR‑125b-2-3p expression value was calculated in comparison to the combination of RNU6B and RNU48 that was previously reported [23,25,30].

#### 2.1.2 The miR-125b-2-3p expression value in hepatocellular carcinoma tissue assessed using high-throughput data

After establishing the expression value of miR-125b-2-3p using in-house RT-qPCR, integrative data were obtained from the miRNA microarray and miRNA sequencing to gauge the different expression levels of miR-125b-2-3p between non-tumorous tissue and HCC tissue using various detection methods. The included datasets were from the Gene Expression Omnibus (GEO) database, the ArrayExpress database, the Sequence Read Archive (SRA) database, ONCOMINE, and The Cancer Genome Atlas (TCGA) database. The keywords used for the article retrieval were ‘hepatocellular carcinoma’ and ‘HCC.’ The inclusion criteria were set as follows: 1) human samples; 2) available miR-125b-2-3p expression data; and 3) sufficient HCC tissue samples and non-cancerous liver controls. The public data-screening process was shown in Supplementary Figures S1–S5. A t test was utilized to examine the expression differences of miR-125b-2-3p between the HCC tissues and the non-tumor tissues via the Statistical Package for the Social Sciences (SPSS), version 22.0, while a ggplot2 package was used to visualize the expression difference. A P value <0.05 indicated statistical significance.

#### 2.1.3 Validation of the expression value of miR-125b-2-3p via integrated analysis

The previous study on miR-125b-2-3p was based on only a small number of cases, and it used nothing outside of its own data to investigate the miR-125b-2-3p expression value[29]. Thus, a comprehensive analysis was carried out using our in-house RT-qPCR, miRNA microarray and miRNA sequencing data to show the overall expression tendency of miR-125b-2-3p between HCC and non-cancerous liver tissues. The expression numbers, expression mean and expression standard deviation of the HCC tissues and non-tumor tissues were extracted for each study. Next, after the inter-batch effect was removed, the standard mean difference (SMD) and the 95% confidence interval (95% CI) were computed using STATA 12.0 software. I^2^ and Chi-square tests were utilized to select either a random effects model or a fixed effects model; a random effects model was used if the study had high heterogeneity (I^2^ > 50% or P < 0.05). In addition, to assess the predictive power of miR-125b-2-3p in liver cancer patients, receiver operating characteristic (ROC) curves and the area under the curves (AUCs) of the ROC curves were appraised using SPSS version 22.0. The potential diagnostic ability of miR-125b-2-3p was credible when the AUC >0.6 and the P value was <0.05. The tp, fp, fn and tn were extracted for each study according to the Yoden Index, and the summary receiver operating characteristic (sROC) was determined for all datasets using STATA 12.0 based on the tp, fp, fn and tn. The summarized AUC was calculated along with the sensitivity and specificity.

#### 2.1.4 An analysis of the clinicopathological and prognostic role of miR-125b-2-3p with in-house RT-qPCR and available public databases

Information regarding any related clinicopathological parameters was screened from the RT-qPCR data and miRNA microarray and miRNA sequencing data from TCGA, SRA and GEO databases to determine the relationship between miR-125b-2-3p and the development of HCC. The clinical parameters could be classified as gender (male OR female), age (>60y OR ≤60y), tumor grade (G1–G2 OR G3–G4), tumor size (T1–T2 OR T3–T4), lymphatic metastasis (N0 OR N1–N3) and hematogenous metastasis (M0 OR M1). A t test was utilized to calculate the relationship between miR-125b-2-3p and each clinicopathological parameter using SPSS version 22.0. An integrative analysis was also performed for several meaningful clinical parameters. Moreover, any prognostic information about miR-125b-2-3p in HCC was collected from multiple datasets, and the hazard ratio (HR) of miR-125b-2-3p was calculated using a Cox proportional hazards model via survival packages in R. Kaplan-Meier (K-M) plots were also drawn for each study. An integrative analysis of the prognostic value of miR-125b-2-3p in HCC was performed.

#### 2.1.5 The correlation between nitidine chloride and miR-125b-2-3p in hepatocellular carcinoma cells

The anti-tumor effects of traditional Chinese medicine in HCC were researched by our team, and we found that nitidine chloride (NC) was an inhibitor of HCC onset and development [31–36]. In our study, miR-125b-2-3p was associated with the progression and prognosis of HCC; however, it was still unknown whether NC could act on miR-125b-2-3p. In this study, an in vivo experiment was carried out in mice to assess the variation tendency of miRNAs and mRNAs in HCC after NC treatment. The mice were purchased from the Shanghai SLAC Laboratory Animal Co., Ltd., and all the experiments were based on the Guide for the Care and Use of Laboratory Animals. All mice had undergone transplants of their SMMC7721 cells (0.2 ml in total, with a density of 1 × 10^7^ cells/ml). When an HCC tumor grew to at least 70 mm^3^ in size, these mice were randomly placed into either the blank control group or the NC-treated group. The nude mice in the control group were injected with normal saline, and the treatment group mice received injections of 7 mg/kg NC. After two weeks, the tumors were extracted from each mouse and stored at −80°C. RNA, including miRNA, was extracted using TRIzol Reagent (Invitrogen, US). A NanoPhotometer® spectrophotometer (IMPLEN, CA, US) was utilized to determine the RNA purity. Then, the RNA integrity was appraised with an RNA Nano 6000 Assay Kit for the Agilent Bioanalyzer 2100 system (Agilent Technologies, CA, US). Next-generation sequencing was used to determine the miRNA and mRNA expression profiles. A Limma Voom package in R software v3.6.1 was used to detect the expression difference of miR-125b-2-3p and its potential targets in the NC-treated group and the control group.

### 2.2 Potential mechanism of miR-125b-2-3p in hepatocellular carcinoma based on its prospective target genes

#### 2.2.1 Screening miR-125b-2-3p target genes

Assessing the final targets of miR-125b-2-3p consisted of three parts. First, in order to rigorously identify the differently expressed genes (DEGs) of HCC, 75 microarrays and high-throughput RNA-sequencing results were obtained from nine different platforms. After extracting the expression of each matrix, the Limma package was used to remove the batch effects of different matrices in the identical platforms. Then, the DEGs of the RNA-sequencing data were calculated using Limma Voom, and microarrays using Limma (logfc >1, adj P < 0.05). After the SMDs of all the genes were calculated, 3,736 up-regulated DEGs were obtained. Second, the putative miR-125b-2-3p target genes were predicted. miRWalk version 2.0 was used for the miRNA target prediction[37]. The targeted genes of miR-125b-2-3p were predicted using 12 prediction software programs included in miRWalk2.0, and those target genes appeared at least three times (n = 5,770) in 12 platforms. Third, considering the genes influenced by NC, we also took the down-regulated genes post-NC treatment into consideration. Altogether, 863 genes were down-regulated after NC treatment. The overlapping genes generated by the above three gene groups were the most likely potential target genes of miR-125b-2-3p in HCC related to the effect of NC.

#### 2.2.2 Potential biological functions and signaling pathways of target genes in hepatocellular carcinoma

Online resource Metascape was used to provide the biofunctional enrichment data required for Gene Ontology (GO) and the Kyoto Encyclopedia of Genes and Genomes (KEGG)[38]. The thresholds for the GO items were set at P < 0.05, the false discovery rate (FDR) at P < 0.05, and the KEGG items at P < 0.05. The top 10 items in the GO enrichment and KEGG pathways were extracted according to P value and visualized using bubble charts and chord diagrams, respectively. A protein-protein interaction (PPI) network was created for these genes that were enriched in the most important KEGG signaling pathways via STRING online tools, and Cytoscape 3.6.1 was utilized to visualize the PPI network. The genes in this PPI network were calculated by Maximum Clique Centrality (MCC) algorithm via cytoHubba, and the top gene in the ranking was taken as the hub gene. Hence, the hub gene was considered as the most important target of miR-125b-2-3p in HCC.

To explore the relationships between hub genes and miR-125b-2-3p in terms of expression level, a correlation analysis was calculated using the expression data extracted from the following four datasets, which included both mRNA and miRNA expression data: the TCGA, GSE22058, GSE57555 and GSE62044 databases. This was done using a Pearson correlation model. A significant correlation existed when P < 0.05. To obtain a comprehensive cognition of the correlation between miR-125b-2-3p and the potential target, the correlation coefficient (r) and the lower 95% CI and upper 95% CI in the four datasets (TCGA, GSE22058, GSE57555 and GSE62044) were used to calculate the SMD via STATA12.0.

#### 2.2.3 The expression value of hub genes in hepatocellular carcinoma

To examine the expression of hub genes in HCC, both mRNA level and protein level were used. For the mRNA level, a total of 75 microarrays and high-throughput RNA-sequencing data were obtained from nine different platforms to detect the mRNA value of hub genes. Then, to determine the overall expression tendency of hub genes in HCC, STATA12.0 was used to calculate the SMD after removing the batch effect. For the protein level, the immunohistochemistry results of hub genes in both HCC and normal liver tissue were gathered from The Human Protein Atlas (THPA) database [39–41].

### 2.3 Statistical analysis

All the statistical analysis and graphs in this study were carried out by SPSS 22.0, STATA 12.0, R software and graphpad7. T test based on SPSS 22.0 was used to compare the differences between two groups. ROC curves were used to test the discrimination value of miR-125b-2-3p. Integrative analyses based on STATA 12.0 were used to get comprehensive information. Pearson’s correlation coefficients were used to calculate the association between miR-125b-2-3p and PRKCA. There was statistical significance when p < 0.05.

## 3. Results

In this study, we found that the expression of miR-125b-2-3p in HCC was evidently lower than in non-tumorous liver tissue based on 1,233 cases of the HCC and 630 cases of the non-HCC controls. Furthermore, lowly-expressed miR-125b-2-3p led to progress and poorer prognosis in HCC. According to the in vivo experiments, miR-125b-2-3p was a potential target of NC and was up-regulated by NC treatment in HCC cells. So miR-125b-2-3p might be a potential predictor and therapeutic target in HCC. MiR-125b-2-3p might active the thyroid hormone signaling pathway to resist HCC according to KEGG analysis. In addition, combined with the in vivo and in silico experiments, PRKCA was the most possible target of miR-125b-2-3p in HCC. In summary, miR-125b-2-3p might play an anticancer role in HCC and is up-regulated by NC.

### 3.1 The differential expression and clinical significance of miR-125b-2-3p in HCC

#### 3.1.1 The expression tendency and clinical significance of miR-125b-2-3p in HCC based on in-house RT-qPCR and public high-throughput data

**Figure 1.**
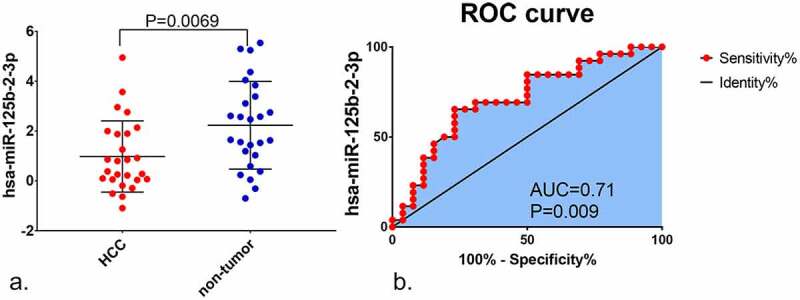
The expression of miR-125b-2-3p in HCC and non-tumor tissue based on in-house RT-qPCR. MiR-125b-2-3p was lowly expressed in HCC tissue compared to non-tumorous liver tissue. (b) The ROC curve of miR-125b-2-3p in HCC

**Figure 2.**
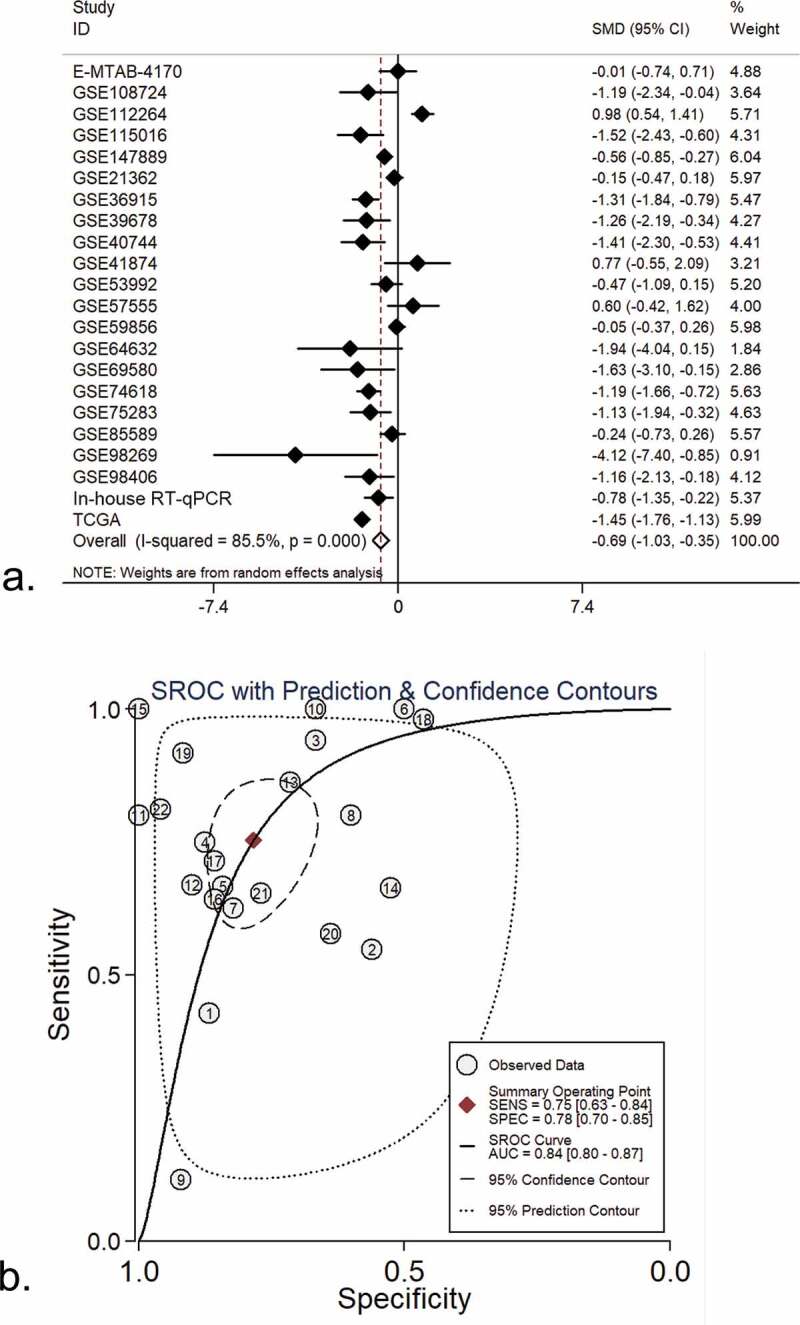
An integrative analysis of miR-125b-2-3p in HCC and non-tumor tissue. (a) Combined with a series of studies, the expression trend of miR-125b-2-3p in HCC and non-tumor tissue was shown by forest plot. A random effects model was used. (b) SROC curves of miR-125b-2-3p based on the integrative studies. The numbers in the figure were: 1) E-MTAB-4170. 2) GSE21362. 3) GSE36915. 4) GSE39678. 5) GSE40744. 6) GSE41874. 7) GSE53992. 8) GSE57555. 9) GSE59856. 10) GSE64632. 11) GSE69580. 12) GSE74618. 13) GSE75283. 14) GSE85589. 15) GSE98269. 16) GSE98406. 17) GSE108724. 18) GSE112264. 19) GSE115016. 20) GSE147889. 21) RT-qPCR. 22) TCGA

Compared to non-tumor liver tissue, miR-125b-2-3p had a remarkably lower expression in HCC, as assessed by in-house RT-qPCR (Figure 1). The expression value of miR-125b-2-3p in each study was visualized via violin plot (Supplementary Figure S6). Our integrative analysis revealed that the SMD was −0.69 (95% CI: −1.03 to −0.35), which implied that a significantly under-expressed level of miR-125b-2-3p existed in 1,233 cases of the HCC and 630 cases of the non-HCC controls (Figure 2a). Due to the high heterogeneity in the preliminary detection (I^2^ = 85.5%, P < 0.001), a random effects model was used. The funnel plot showed no publication bias (Supplementary Figure S7A). After deleting the high heterogeneity studies (Supplementary Figure S7B), the secondary SMD was −0.98 (95% CI: −1.24 to −0.71), and the trend of low miR-125b-2-3p expression in HCC was more obvious (Supplementary Figure S8A). There was no publication bias in our study after removing the high-heterogeneity studies (Supplementary Figure S8B).

The ROC curves were utilized to assess the predictive significance of miR-125b-2-3p in HCC for each study (Supplementary Figure S9). The AUC of the SROC curve was 0.84 (95% CI: 0.80–0.87, Figure 2b), the sensitivity was 0.76 (range: 0.64–0.86) and the specificity was 0.79 (0.71–0.86, Supplementary Figure S10). These findings indicated that downregulation of miR-125b-2-3p can be used as a factor to discern HCC from non-HCC liver tissues.

#### 3.1.2 The clinicopathological significance of miR-125b-2-3p in HCC

**Figure 3.**
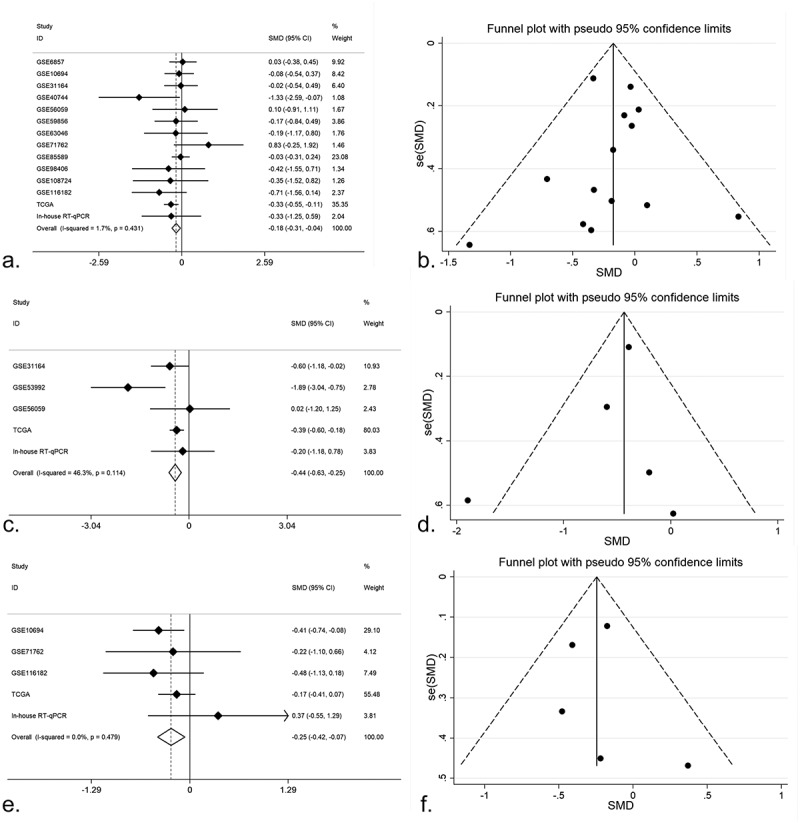
(a) An integrative analysis of miR-125b-2-3p based on gender group. MiR-125b-2-3p was lowly-expressed in female group. (b) Funnel plot of gender group. (c) An integrative analysis of miR-125b-2-3p based on tumor grade. MiR-125b-2-3p was lowly-expressed in poorly differentiated HCC tissue. (d) Funnel plot of grade group. (e) An integrative analysis of miR-125b-2-3p based on TNM stage. MiR-125b-2-3p was lowly-expressed in advanced TNM stages. (f) Funnel plot of TNM stage group

We browsed all the included datasets and extracted the clinical parameter information of HCC. The expression of miR-125b-2-3p in various clinical parameters was determined (Supplementary Table S1). In the clinical parameter analysis based on TCGA, gender and tumor grade were highly associated with miR-125b-2-3p, while miR-125b-2-3p had a lower expression level in females than in males (P = 0.006; Supplementary Figure S11A) and was only slightly expressed in poorly differentiated HCC tissue (G3–G4) (P < 0.001; Supplementary Figure S11B, Supplementary Table S2). Tumor grade and gender information were obtained from in-house RT-qPCR and all available miRNA-array and miRNA-seq datasets (Supplementary Figures S2–S3). Total 179 poorly differentiated samples and 315 highly differentiated samples of HCC were obtained, and total 310 male patients and 1003 female patients were included. The results of an integrative analysis revealed that miR-125b-2-3p was expressed at lower levels in females as well as in poorly differentiated HCC tissue (Figure 3(a–Figure 3d)). Furthermore, a conflicting result was obtained from the TNM stage analysis between in-house RT-qPCR data and TCGA RNA-sequencing data. In the TCGA data, miR-125b-2-3p was highly expressed in the early stages of HCC, but it was expressed at lower levels in advanced stages of HCC according to the RT-qPCR data. To explain this contradictory phenomenon, information regarding the TNM stage was extracted from multiple datasets (Supplementary Figure S4). The result of an integrative analysis indicated that miR-125b-2-3p was expressed at lower levels in advanced TNM stages based on the 192 advanced cases and the 412 early cases (Figure 3(e–Figure 3f)).

#### 3.1.3 The prognostic value of miR-125b-2-3p in HCC

**Figure 4.**
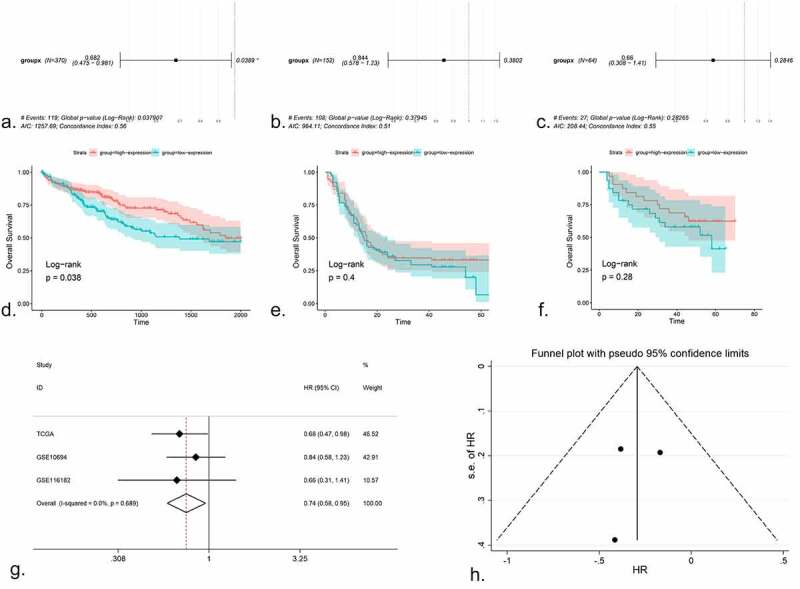
The prognostic value of miR-125b-2-3p in HCC, the COX regression, K-M plots and SMD were performed. (a-c) COX regression analysis of miR-125b-2-3p in TCGA, GSE10694 and GSE116182. (d-f) K-M plots were performed for TCGA, GSE10694 and GSE116182 respectively. (g) The forest plot was drawn based on the HR. The experimental group was high-expressed miR-125b-2-3p group and the high-expressed miR-125b-2-3p was indicated a great prognosis in HCC. (h) the funnel plot of HR

The available prognostic information was obtained from TCGA, GSE10694 and GSE116182 (Supplementary Figure S5). After Cox regression testing, the HR of the high expression group in TCGA was 0.682, with a 95% CI: 0.475–0.981 and P = 0.038 (Figure 4a). In GSE10694, the HR was 0.844 (95% CI: 0.578–1.23, P = 0.38; Figure 4b). In GSE116182, the HR was 0.66 (95% CI: 0.308–1.41, P = 0.28; Figure 4c). K-M plots were created for all studies (Figure 4(d–Figure 4f)). It was evident that the HR of the high expression group in each dataset was <1; thus, a higher expression of miR-125b-2-3p was associated with a better prognosis. However, only the TCGA data had statistical significance; thus, an integrative analysis was performed using the HR of the three datasets, including 533 HCC cases, which indicated that miR-125b-2-3p could be a protective indicator of the survival of HCC (Figure 4(g–Figure 4h)).

### 3.2 Potential biological mechanisms of miR-125b-2-3p in HCC

#### 3.2.1 Enrichment analysis of overlapping genes

**Figure 5.**
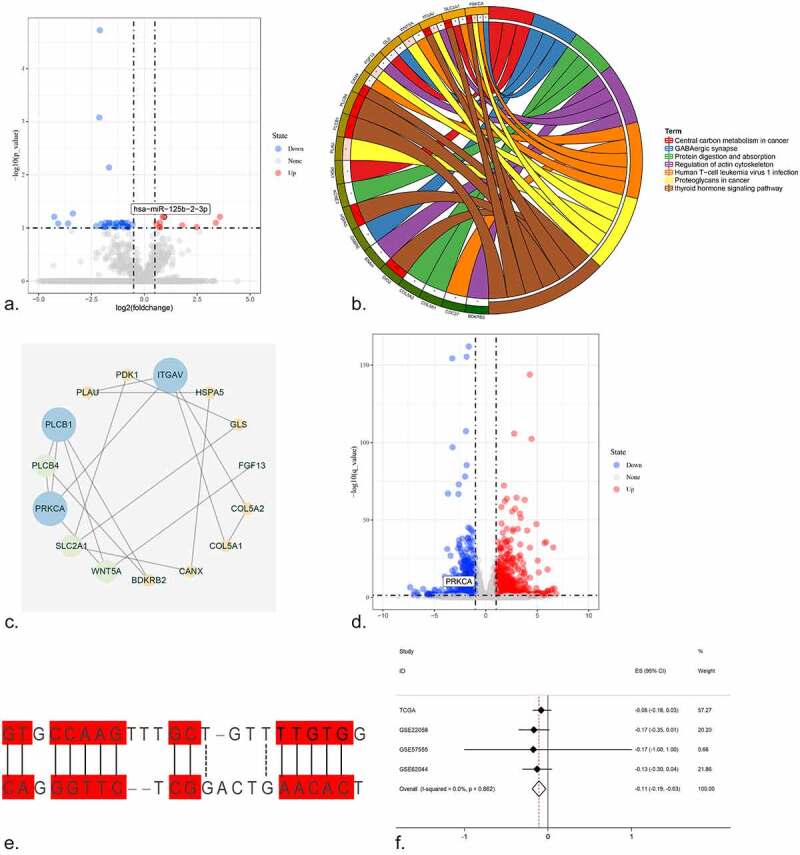
(a) The volcano plot of miRNAs in HCC cell line. After NC treatment, the expression of miR-125b-2-3p was higher than in control group. (b) The KEGG pathway analysis was performed for the target genes of miR-125b-2-3p. Different colors indicated different terms. (c) The PPI network for all the genes enriched in KEGG pathways. (d) The volcano plot of mRNAs in HCC cell line. Hub gene PRKCA was low-expressed in HCC cell line after NC treatment. (e) The complementary sequence between miR-125b-2-3p and PRKCA. (f) An integrative analysis of correlation analysis between miR-125b-2-3p and PRKCA

A volcano plot was drawn for all miRNAs in the control and NC-treated HCC tissues, and miR-125b-2-3p was highly expressed in the NC-treated tissue (Figure 5a). The expression value of miR-125b-2-3p in the control tumor tissue and the NC-treated tissue was visualized using a boxplot, which showed that the expression level of miR-125b-2-3p was markedly increased following NC treatment compared to the control liver tumor tissue (Supplementary Figure S12A, Supplementary Figure S12B). In total, 80 overlapping genes were obtained after intersecting the targeted genes of miR-125b-2-3p, HCC over-expressed genes, and downregulated genes after NC-treated HCC cell lines (Supplementary Figure S13). The top three items produced by the GO analysis were regulation of embryonic development, focal adhesion and calcium ion binding. The top 10 items returned by the GO analysis were visualized using a bubble chart, where the size of the bubble indicated the enrichment gene counts and the color illustrated the P value (Supplementary Figure S14).

In the KEGG pathway analysis, the top three pathways were the thyroid hormone signaling pathway, proteoglycans in cancer, and central carbon metabolism in cancer. The important KEGG pathways and enrichment genes were reflected in the chord diagram; different color blocks indicated each pathway, while genes and pathways were connected by lines (Figure 5b). The thyroid hormone signaling pathway was so important that the enrichment genes were used for the PPI network analysis (Figure 5c). The hub gene for the thyroid hormone signaling pathway was protein kinase C, alpha (PRKCA). The expression of all the mRNAs in the control and NC-treated HCC cell lines was shown via a volcano plot, and interestingly, that PRKCA was low-expressed in HCC after NC treatment (Figure 5d). In addition, the complementary sequence of PRKCA and miR-125b-2-3p was confirmed (Figure 5e).

A Pearson correlation analysis revealed the relationship of the hub target PRKCA and miR-125b-2-3p, and a scatter plot was performed for the four datasets to reveal the correlation tendency of PRKCA and miR-125b-2-3p (Supplementary Figure S15). In addition, the integrative analysis of the correlation result showed that a negative correlation did exist between PRKCA and miR-125b-2-3p (figure 5f).

**Figure 6.**
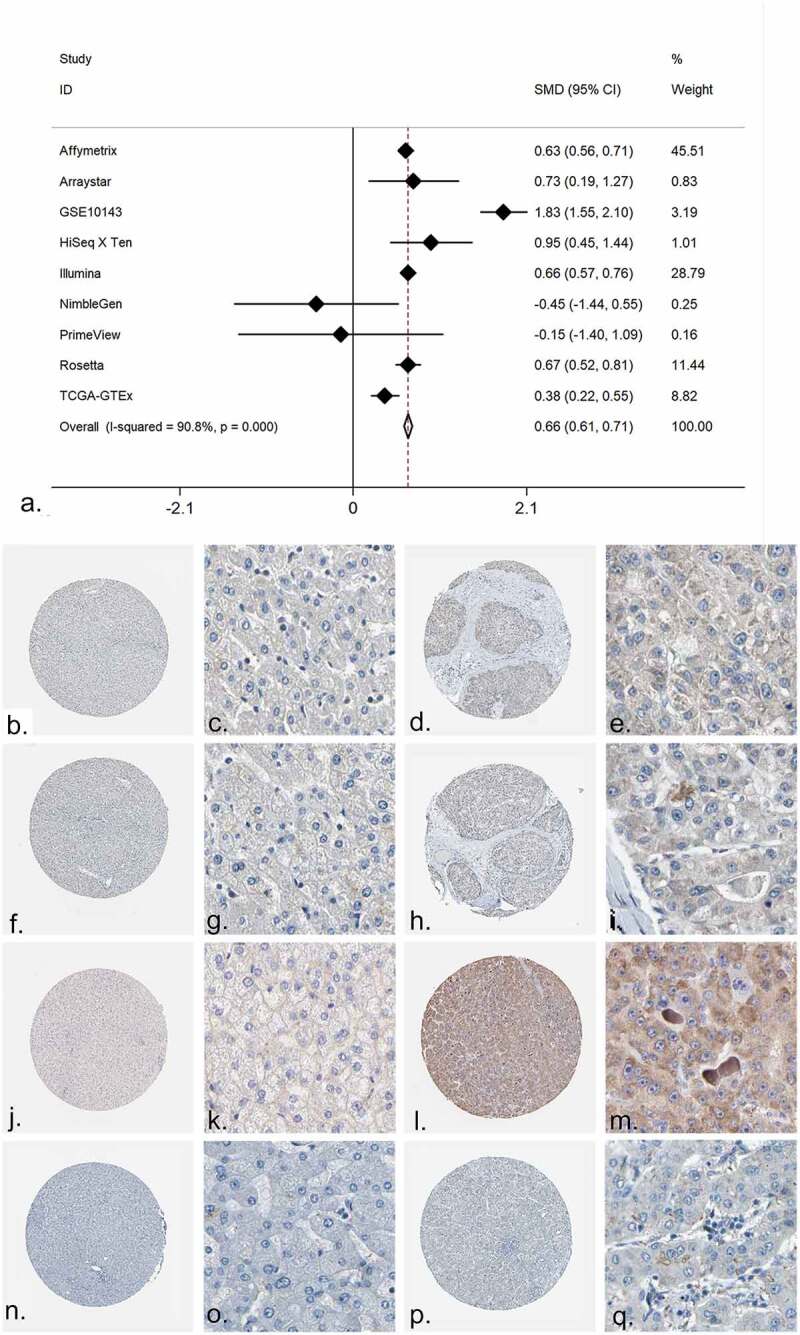
The expression of PRKCA in HCC. (a) An integrative analysis of PRKCA expression in HCC. (b-q) The immunohistochemistry profiles of PRKCA in HCC and normal liver tissue based on multiple antibodies. B-E): PRKCA stained in normal liver tissue and HCC by antibody HPA006563. B: ×40, C: ×400, D: ×40, E:×400. F-I): PRKCA stained in normal liver tissue and HCC by antibody HPA006564. F: ×40, G: ×400, H: ×40, I:×400. J-M): PRKCA stained in normal liver tissue and HCC by antibody CAB003844. J: ×40, K: ×400, L: ×40, M:×400. N-Q): PRKCA stained in normal liver tissue and HCC by antibody CAB016290

The expression value of PRKCA in both mRNA level and protein level was detected. The mRNA level was shown by forest plot, and the expression of PRKCA was markedly increased in the HCC compared to the non-cancerous liver tissue (Figure 6a, Supplementary Figure S16). The immunohistochemistry detections were also collected; a total of four antibodies were utilized to detect the protein level of PRKCA in HCC. As shown in Figure 6(b-Figure 6q), the protein level of PRKCA in HCC was higher than in normal liver tissue. The results of the t test for the four antibodies were shown via scatter diagrams (Supplementary Figure S17).

## 4. Discussion

Few studies have investigated miR-125b-2-3p in liver cancer; only Shi et al. reported an association between miR-125b-2-3p expression and HCC. Shi et al. used 12 paired HCC and non-tumorous tissue samples to detect the expression level of miR-125b-2-3p in HCC and found that it was low[29]. However, these researchers only used their own samples and data; multiple cohort samples to assess reliability were still lacking. Therefore, all the available data on miR-125b-2-3p were collected and integrated from GEO, TCGA, ArrayExpress and in-house RT-qPCR to thoroughly investigate the expression value and clinical significance of miR-125b-2-3p in HCC, and 630 non-tumor liver samples and 1,233 HCC samples were obtained. In our study, the expression of miR-125b-2-3p in HCC demonstrated a decreasing trend compared to non-cancerous tissue, and different expression levels of miR-125b-2-3p existed according to gender, tumor grade and TNM stage. miR-125b-2-3p also had a significant prognostic value in HCC. Furthermore, miR-125b-2-3p likely played a significant role in the onset and development of HCC.

To obtain a comprehensive picture of miR-125b-2-3p in HCC, multiple datasets (including in-house RT-qPCR, miRNA microarray and miRNA sequencing) were utilized to detect the expression value of miR-125b-2-3p. Various statistical methods, including the t test, SMD and SROC curve, were used in the analysis. The results of the RT-qPCR data based on 26 pairs of samples revealed that the expression value of miR-125b-2-3p was 3.66 in HCC and 9.61 in non-cancerous tissue, which was 1.63-fold higher than the HCC tissue. To verify this finding, we mined data from high-throughput datasets. The preliminary SMD was −0.71 based on 1,233 HCC samples and 630 non-tumor samples. After removing the heterogeneity studies, the SMD was −0.98; this trend of low expression was remarkable. Moreover, the SROC result indicated that miR-125b-2-3p could be viewed as a predictive factor in HCC. Hence, the downregulation of miR-125b-2-3p could contribute to the onset of HCC.

Because the previous study lacked a clinical parameter analysis, our study was the first to investigate the relationship between the progression and prognosis of HCC and miR-125b-2-3p. miR-125b-2-3p had a markedly low expression level in females and in poorly differentiated HCC tissue in the TCGA data. In addition, tumor grade was an important index that reflected tumor progression. An integrative analysis of the tumor grades was carried out, and the SMD showed that a low expression of miR-125b-2-3p indicated a poorly differentiated outcome for HCC. miR-125b-2-3p had a lower expression level in advanced-stage HCC tissue, and the miR-125b-2-3p expression value was negatively correlated with the TNM stage of HCC. In addition, according to the Cox regression test and forest plot of HR, a high expression level of miR-125b-2-3p was related to a better prognosis in HCC. Therefore, miR-125b-2-3p could be considered a tumor suppressor and a protective indicator for HCC.

Our team previously found that NC was a tumor inhibitor in HCC [31–36], and in this study, miR-125b-2-3p could be considered a protective factor in HCC. Thus, an in vivo experiment was designed to explore the relationship between NC and miR-125b-2-3p. The expression level of miR-125b-2-3p in NC-treated tissue increased clearly than the level recorded in the control group, which indicated its potential therapeutic effect on HCC with the use of NC.

To thoroughly explore the molecular mechanisms and molecular effects of miR-125b-2-3p in HCC, three parts of genes were considered to gather the potential targets of miR-125b-2-3p in HCC under the treatment of NC. Hence, the hub gene protein kinase C, alpha (PRKCA) was determined using a PPI network analysis to structure a regulatory axis of HCC. PRKCA has been reported to be associated with the progression of a variety of cancers, including lung cancer, chordoid gliomas and esophageal carcinoma [42–45]. However, only two groups have reported the correlations between PRKCA and HCC by far [46,47]. Lei et al. have reported that the DNAJB1-PRKCA fusion transcript could cause increased cAMP-dependent protein kinase activity in fibrolamellar HCC, a specific subtype of children liver cancer, and PKA fusion protein played an oncogenic function in HCC cells[46]. Yang et al recalculated the data from GSE69164, GSE63863 and GSE55758 and found that PRKCA was dysregulated in HCC tissues[47]. To confirm the target relationship between PRKCA and miR-125b-2-3p, both the analyses of correlation and expression level of PRKCA in HCC were carried out. The integrative results of the Pearson correlation analysis showed a significantly negative correlation between PRKCA and miR-125b-2-3p, and the high mRNA and protein levels of PRKCA in HCC also confirmed the targeting relationship of miR-125b-2-3p and PRKCA. Hence, the regulatory axis of miR-125b-2-3p-PRKCA might play an important role in HCC. NC can inhibit the expression of PRKCA by up-regulating the expression of miR-125b-2-3p and then play an anti-cancer role.

However, some shortcomings existed in our study. Although the targeted hub gene PRKCA was strongly considered to be the target gene of miR-125b-2-3p, a dual-luciferase experimental confirmation was absent. Additionally, further in vivo or in vitro studies are needed to access the regulatory axis of miR-125b-2-3p-PRKCA in HCC.

## 5. Conclusions

This was the first study to promulgate the clinical significance of miR-125b-2-3p in HCC. The results showed a lower-expressed level of miR-125b-2-3p that was obvious in the HCC tissue compared with the non-tumor tissue, and the lower expression of miR-125b-2-3p indicated poor progression and prognosis in HCC. The miR-125b-2-3p–PRKCA axis could be regulated by the natural bioactive phytochemical alkaloid, NC, in HCC cells. NC can up-regulates the expression of miR-125b-2-3p to play an anti-cancer effect in HCC, which offers new directions and options for the comprehensive treatment of HCC, especially the use of traditional Chinese medicine, to assist in cancer treatment.

## Supplementary Material

Supplemental Material

## Highlights

1. miR-125b-2-3p is evidently down-regulated in 1863 cases of HCC tissues by multiple detecting methods.

2. Lower expression of miR-125b-2-3p accelerates the progression of HCC.

3. miR-125b-2-3p acts as a protective factor for the survival of HCC.

4. Nitidine chloride, a traditional Chinese medicine can increase miR-125b-2-3p level in HCC *in vivo*.

5. miR-125b-2-3p played its protective role in HCC through different targets and pathways, and a potential target PRKCA is worthy of further study.
