# Supplementary material for: Down-regulation of microRNA-125b-2-3p is a risk factor for a poor prognosis in hepatocellular carcinoma
Source: Bioengineered. 2021 May 5;12(1):1627–41. doi: 10.1080/21655979.2021.1921549 (PMC8806266; doi:10.1080/21655979.2021.1921549)
Supplement: Supplemental Material [file KBIE_A_1921549_SM6832.docx]

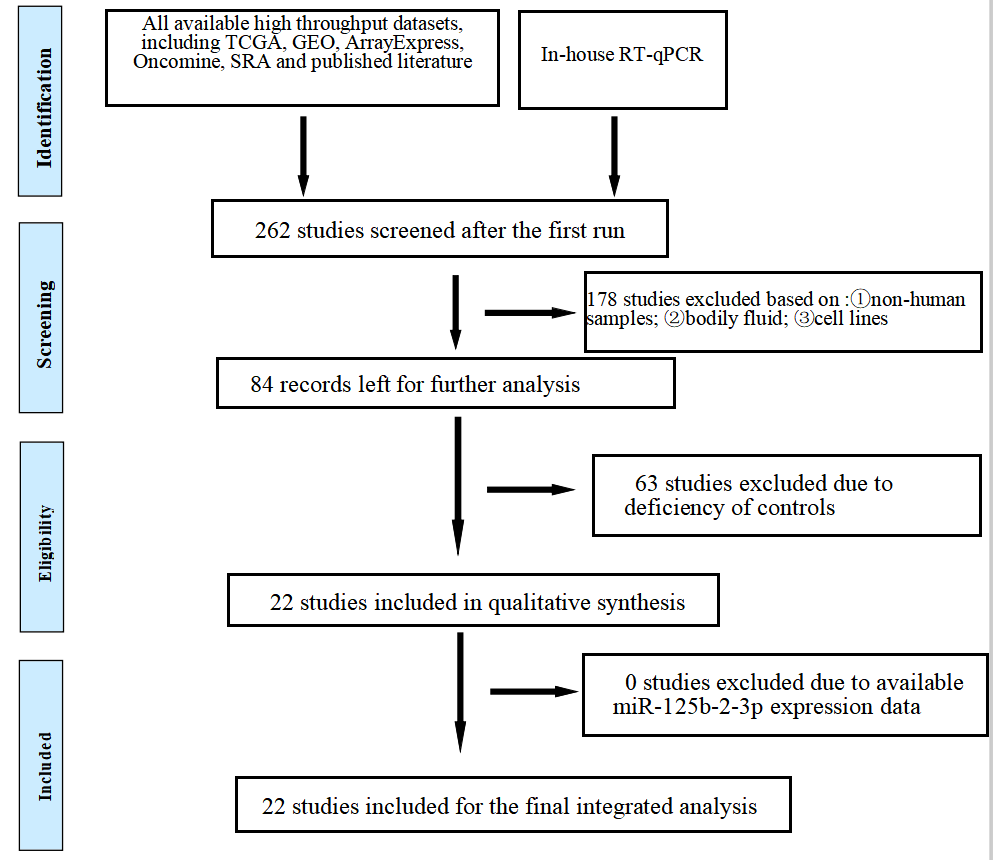


Supplementary figure 1 flow chart of screening expression data.


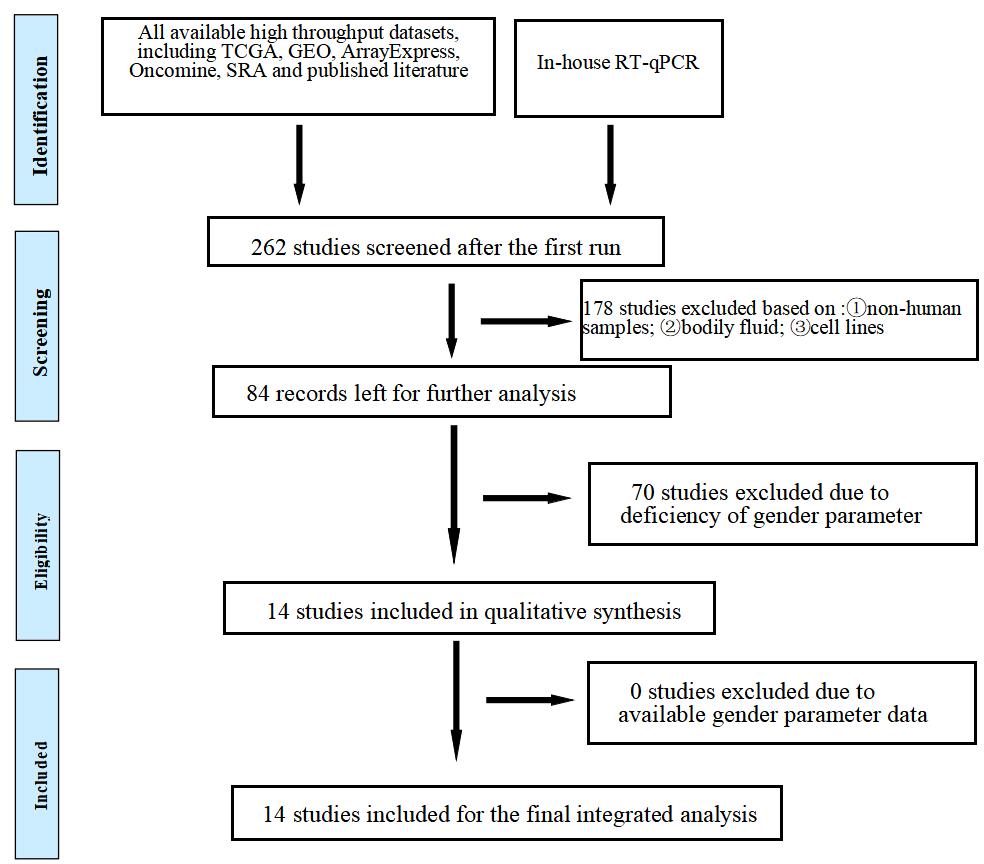


Supplementary figure 2 flow chart of screening gender parameter data.


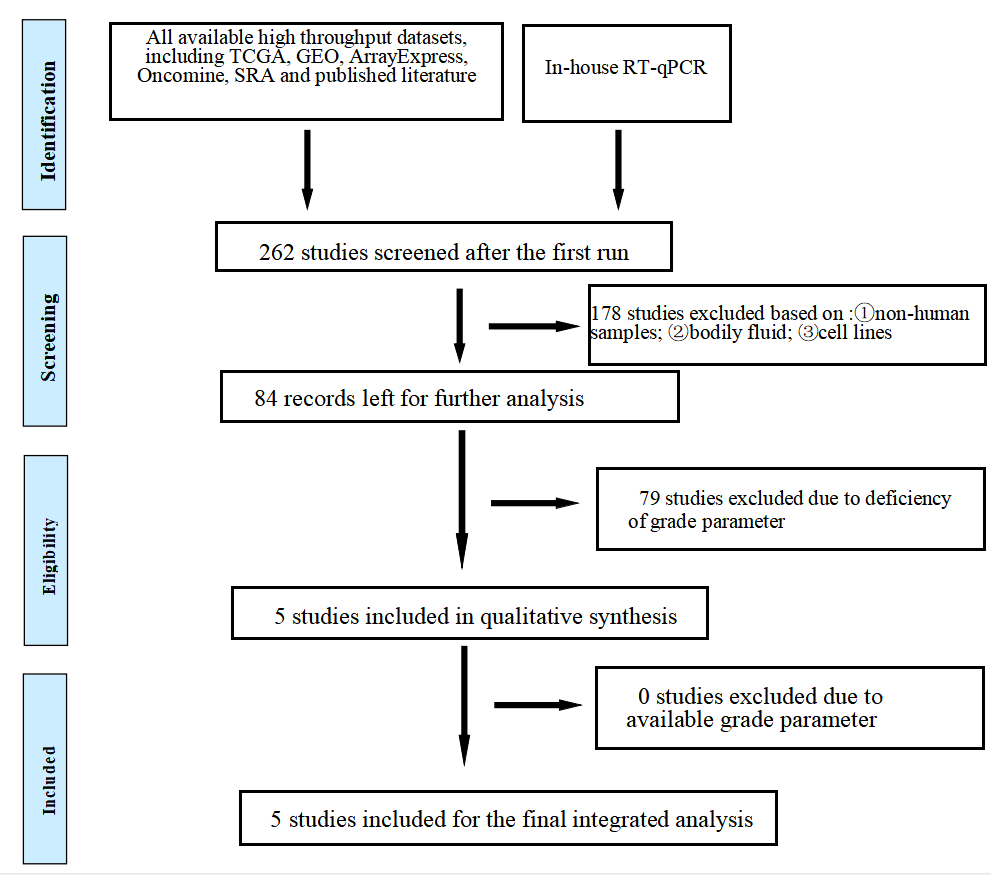


Supplementary figure 3 flow chart of screening tumor grade data.


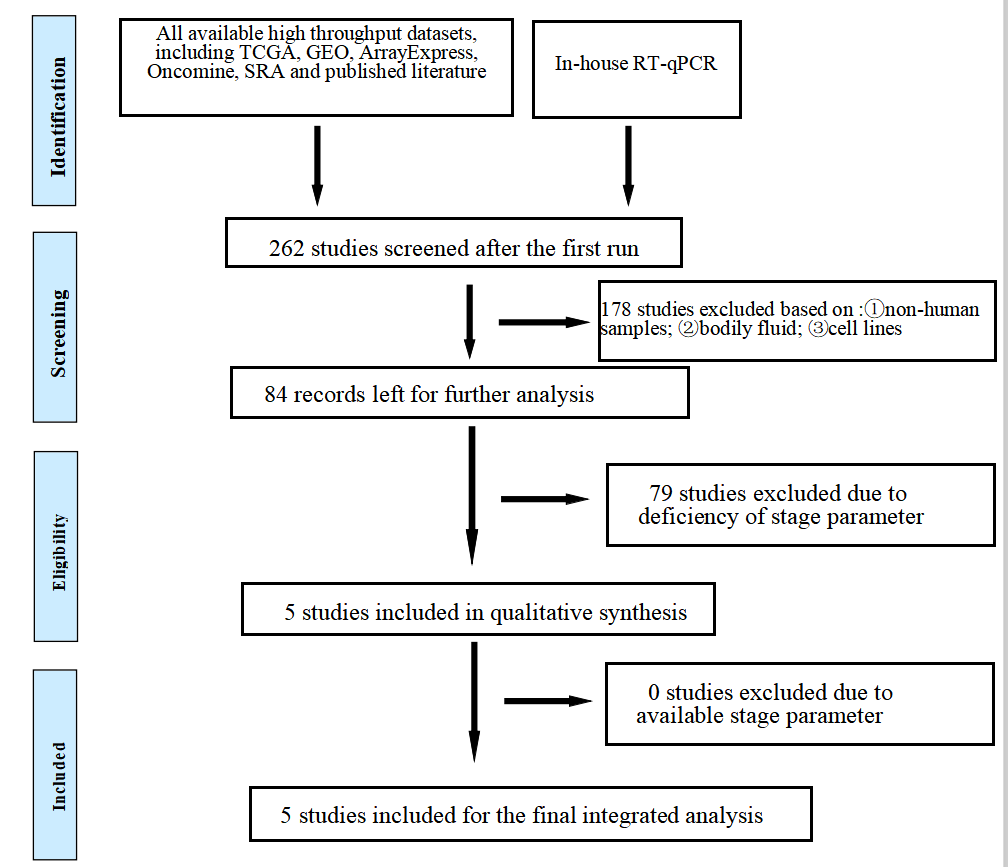


Supplementary figure 4 Flow chart of screening TNM stage data.


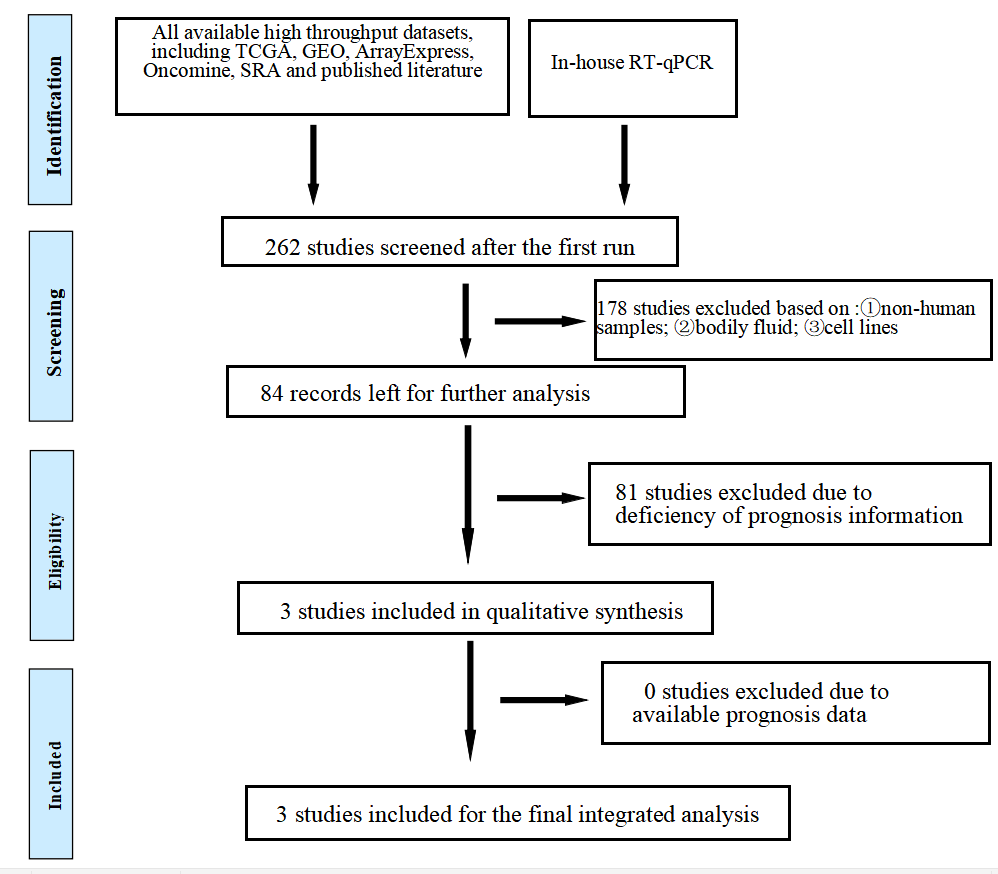


Supplementary figure 5 flow chart of screening prognosis data.


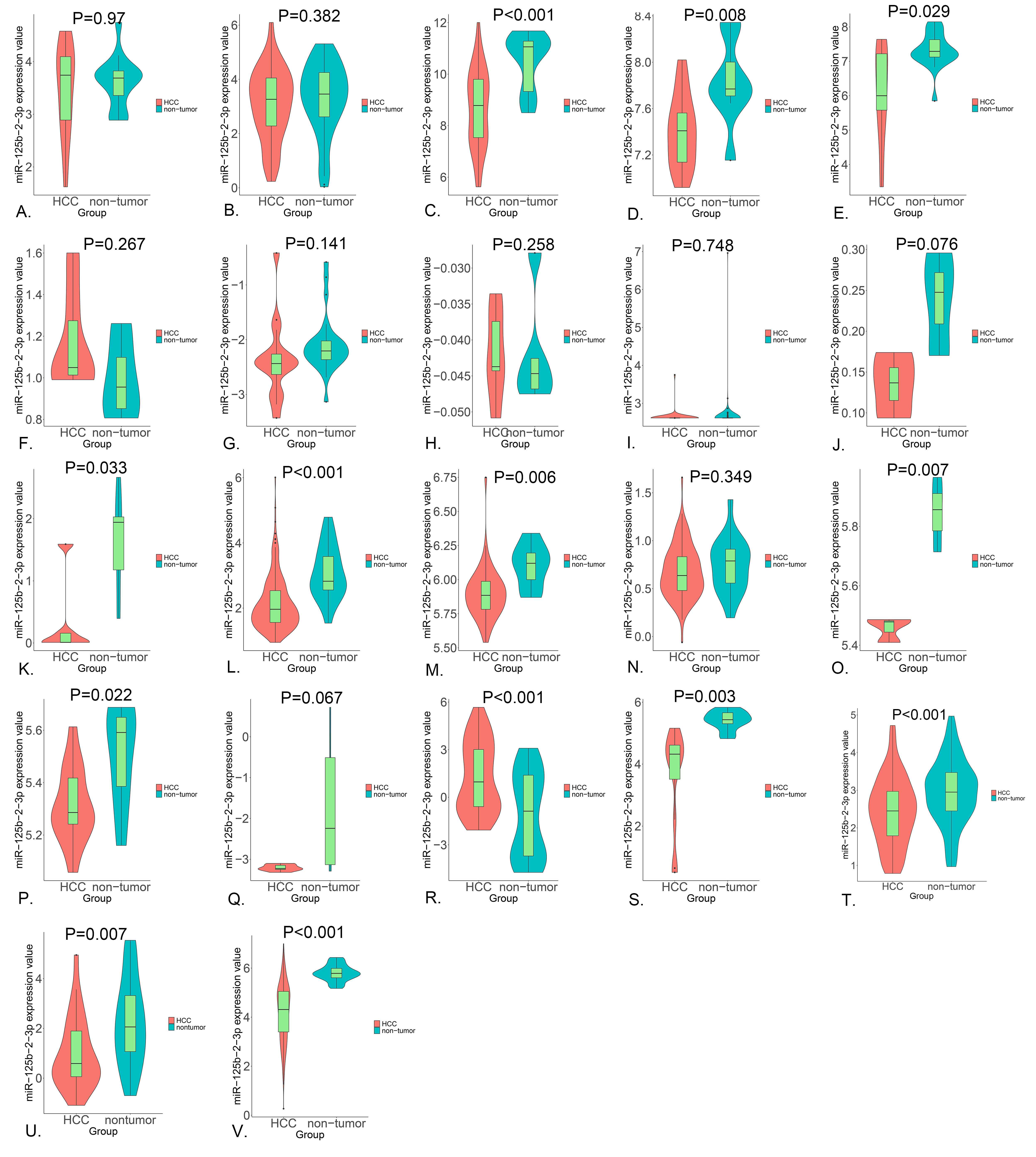


Supplementary figure 6 The expression of miR-125b-2-3p in each study. The width of violin was reflected the samples enrichment degree and the box plot was reflected the mean value and quartile.

(A)E-MTAB-4170. (B)GSE21362. (C)GSE36915 (D)GSE39678. (E)GSE40744. (F)GSE41874. (G)GSE53992. (H)GSE57555. (I)GSE59856. (J)GSE64632. (K)GSE69580. (L)GSE74618. (M)GSE75283. (N)GSE85589. (O)GSE98269. (P)GSE98406. (Q)GSE108724. (R)GSE112264. (S)GSE115016. (T)GSE147889. (U)RT-qPCR. (V)TCGA


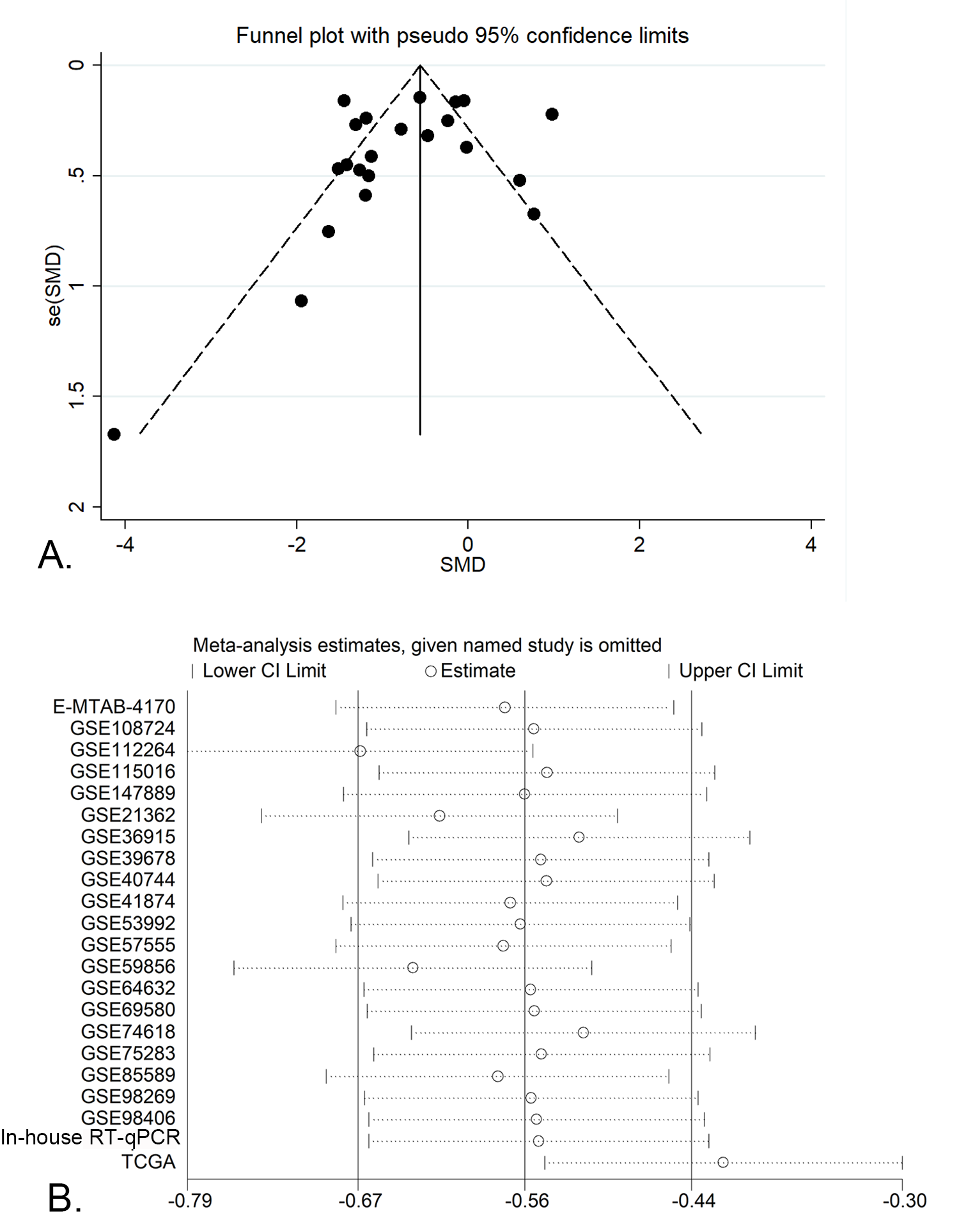


Supplementary figure 7 The assessment results of publication bias and heterogeneity of integrative analysis for miR-125b-2-3p in HCC.

1. Funnel plot was performed to evaluate the publication bias. (B) Sensitivity analysis was used to screen high heterogeneity data.


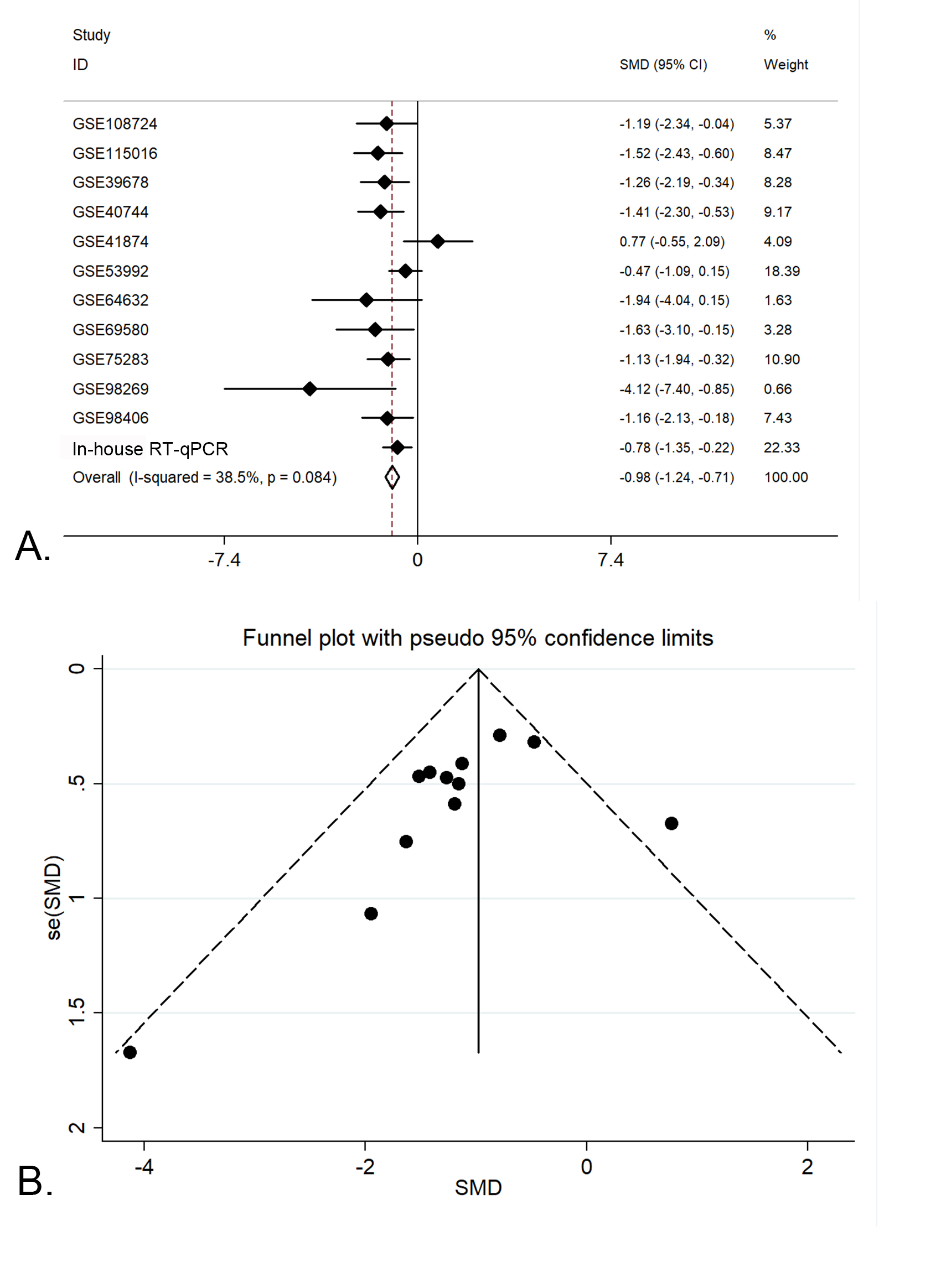


Supplementary figure 8 Further integrative analysis of integrative studies after excluding the high heterogeneity studies.

(A) A random effect model was used in the further analysis. (B) Funnel plot of included studies removing the high heterogeneity studies.


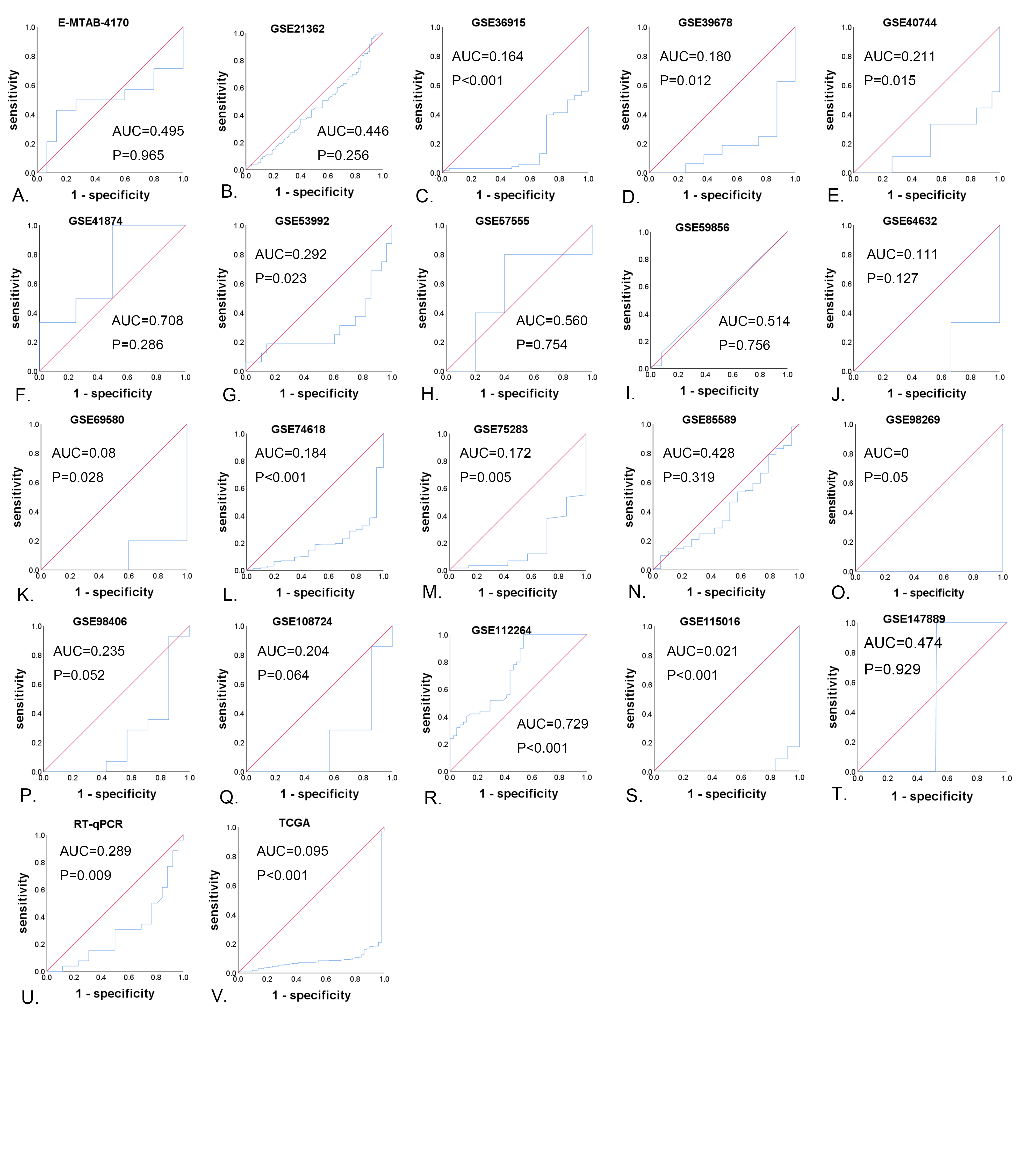


Supplementary figure 9 The roc curves were performed for each study.

(A)E-MTAB-4170. (B)GSE21362. (C)GSE36915 (D)GSE39678. (E)GSE40744. (F)GSE41874. (G)GSE53992. (H)GSE57555. (I)GSE59856. (J)GSE64632. (K)GSE69580. (L)GSE74618. (M)GSE75283. (N)GSE85589. (O)GSE98269. (P)GSE98406. (Q)GSE108724. (R)GSE112264. (S)GSE115016. (T)GSE147889. (U)RT-qPCR. (V)TCGA


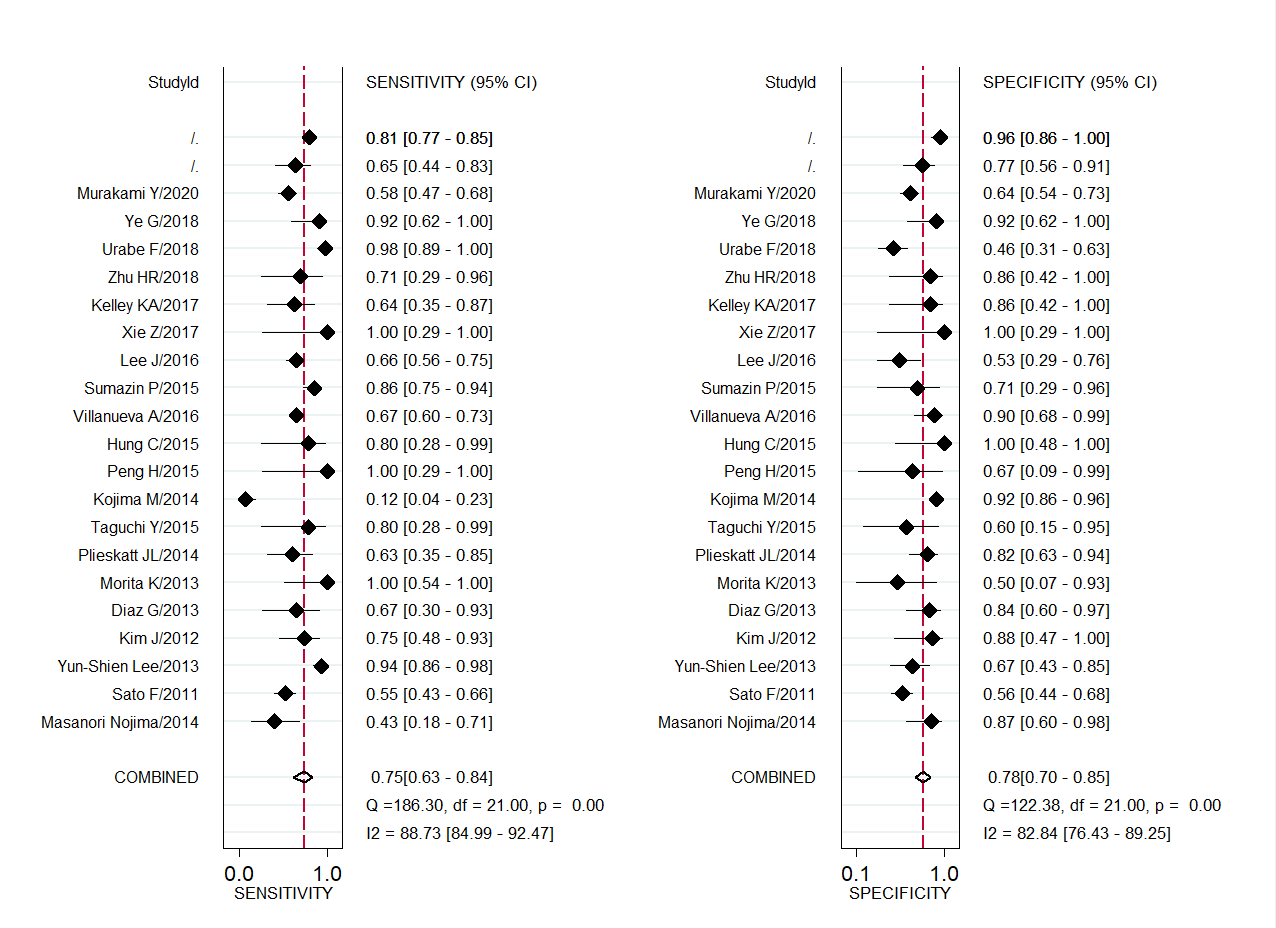


Supplementary figure 10 The sensitivity and specificity of SROC curve.


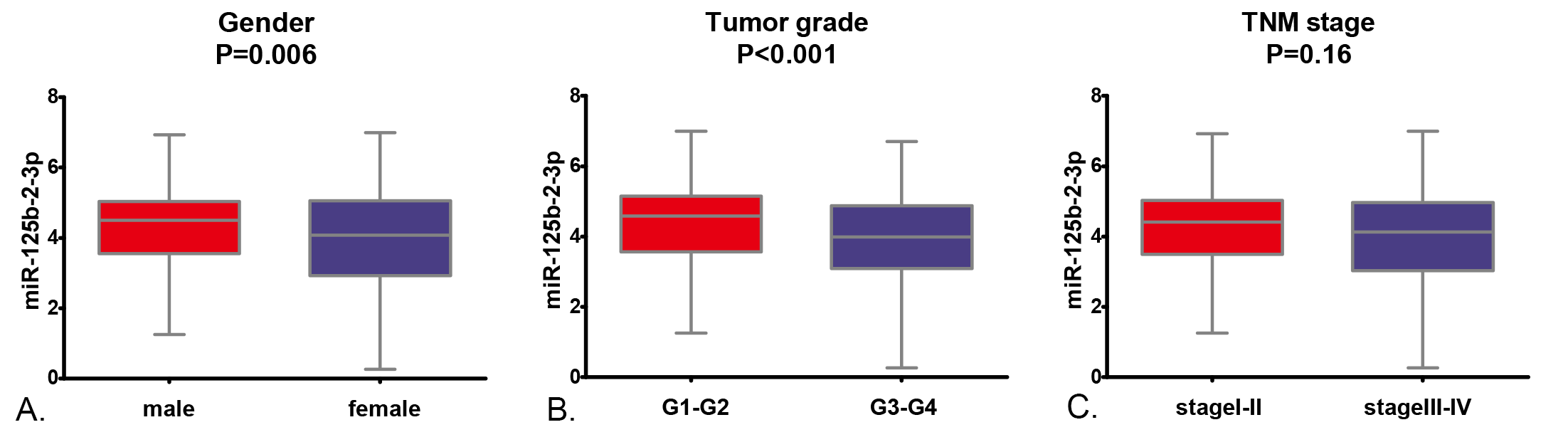


Supplementary figure 11 The expression level of miR-125b-2-3p in two meaningful pathological parameters based on TCGA.

(A) Gender. (B) Tumor grade. (C) TNM stage.


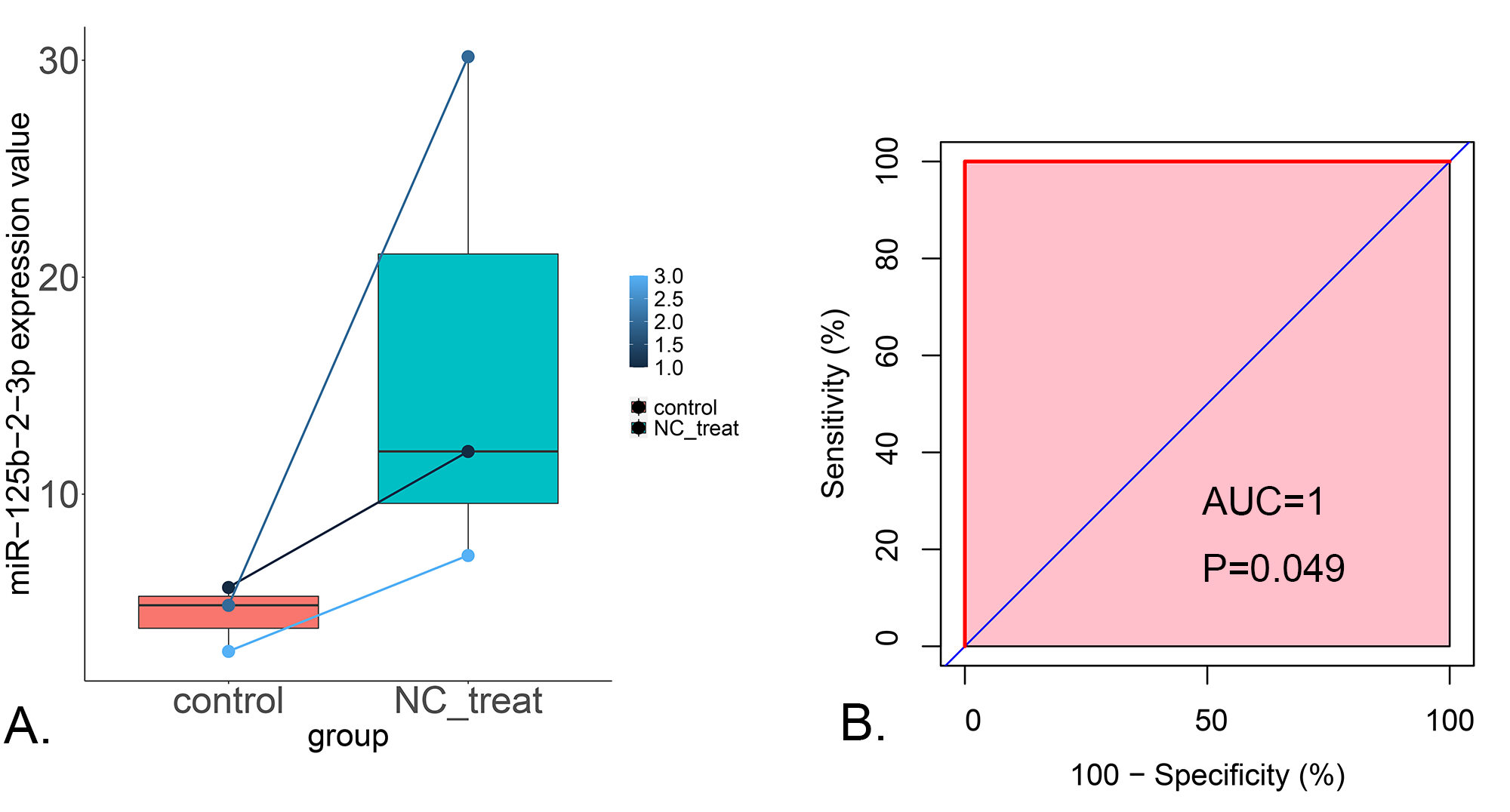


Supplementary figure 12 The expression of miR-125b-2-3p in control HCC tissue and NC-treated HCC tissue. (A) The scatter plot showed that miR-125b-2-3p was high-expressed in each HCC samples after NC treatment. (B) The ROC curve for miR-125b-2-3p.


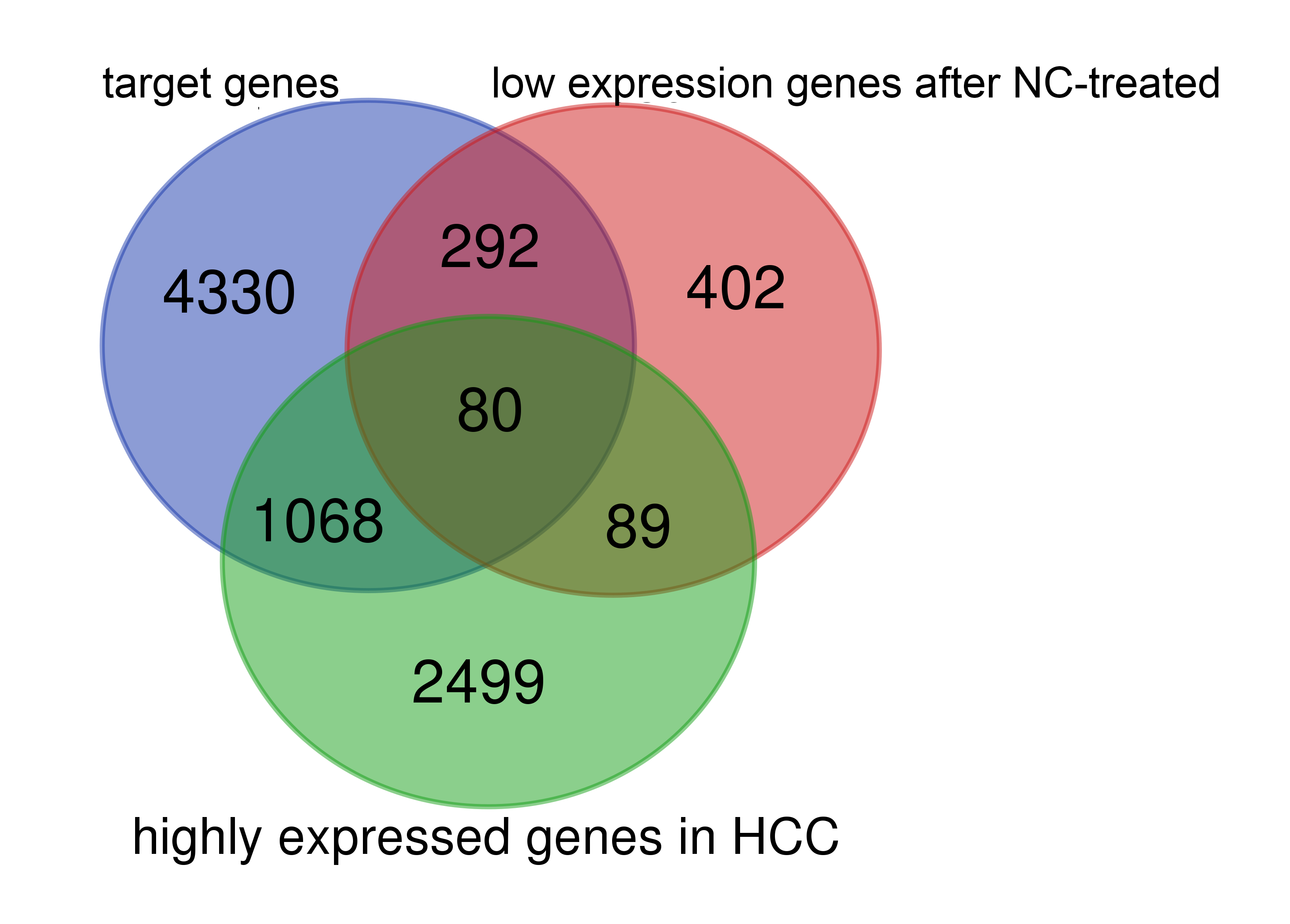


Supplementary figure 13 The venn plot enrolled three group mRNAs, including the target genes of miR-125b-2-3p, the highly expressed genes in HCC and the lowly expressed genes in HCC cell line using NC treatment.


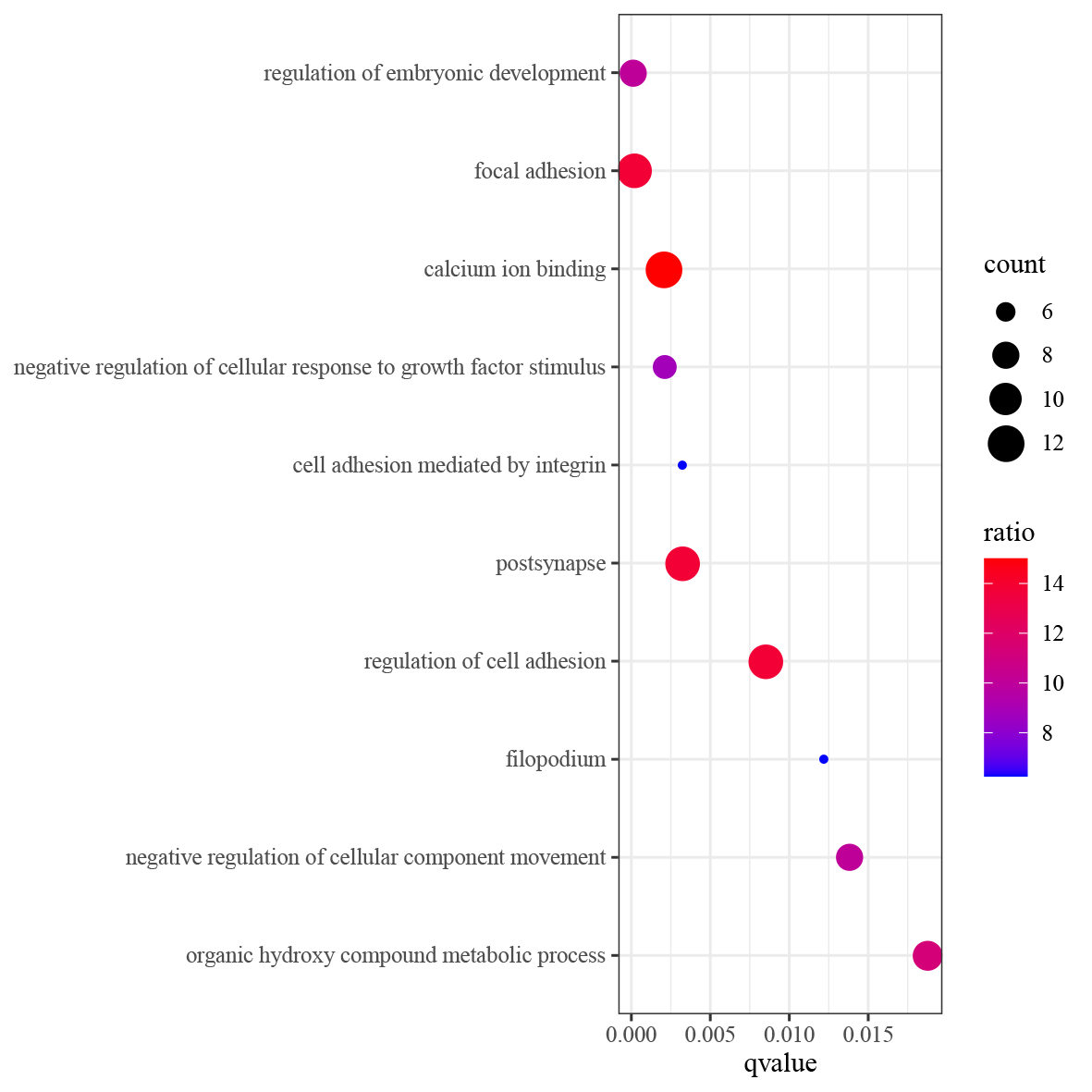


Supplementary figure 14 The GO analysis of intersection genes. The size of the points indicated the enrichment gene counts, and the color indicated the P value.


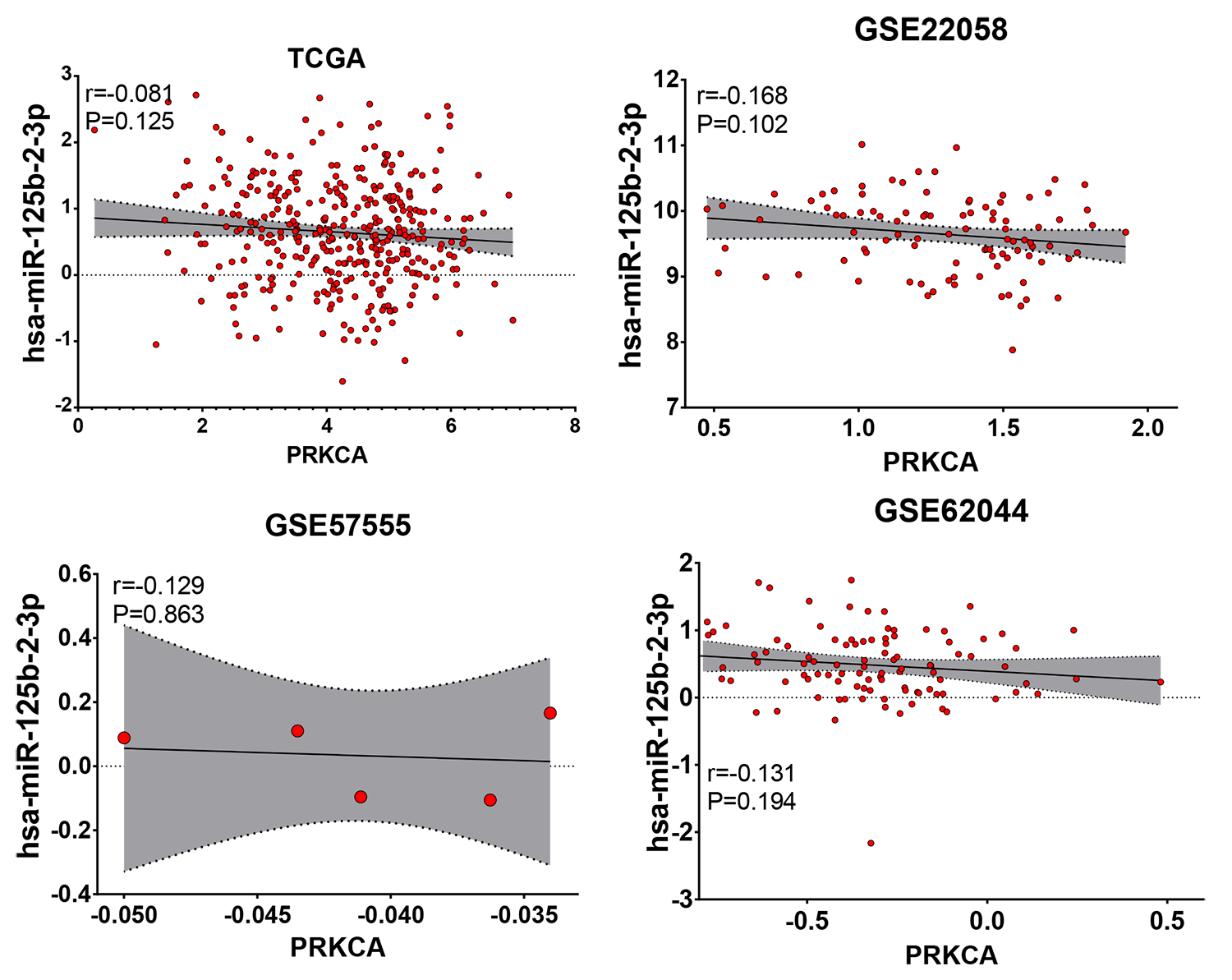


Supplementary figure 15 Correlation analysis between miR-125b-2-3p and PRKCA in HCC. In our including studies, only TCGA, GSE22058, GSE57555 and GSE62044 had the corresponding samples of microRNA and mRNA.


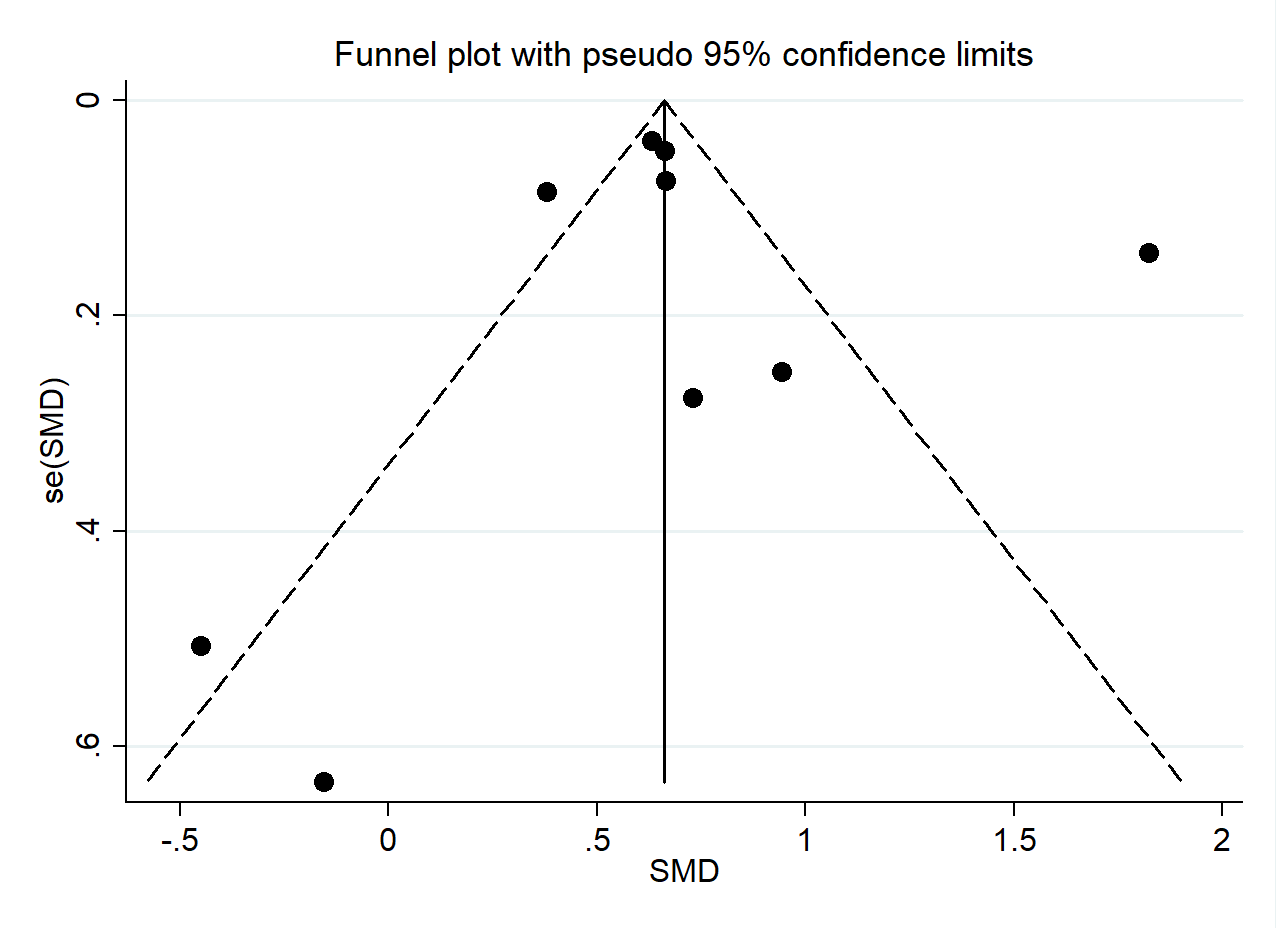


Supplementary figure 16 Funnel plot of integrative analysis for PRKCA in HCC.


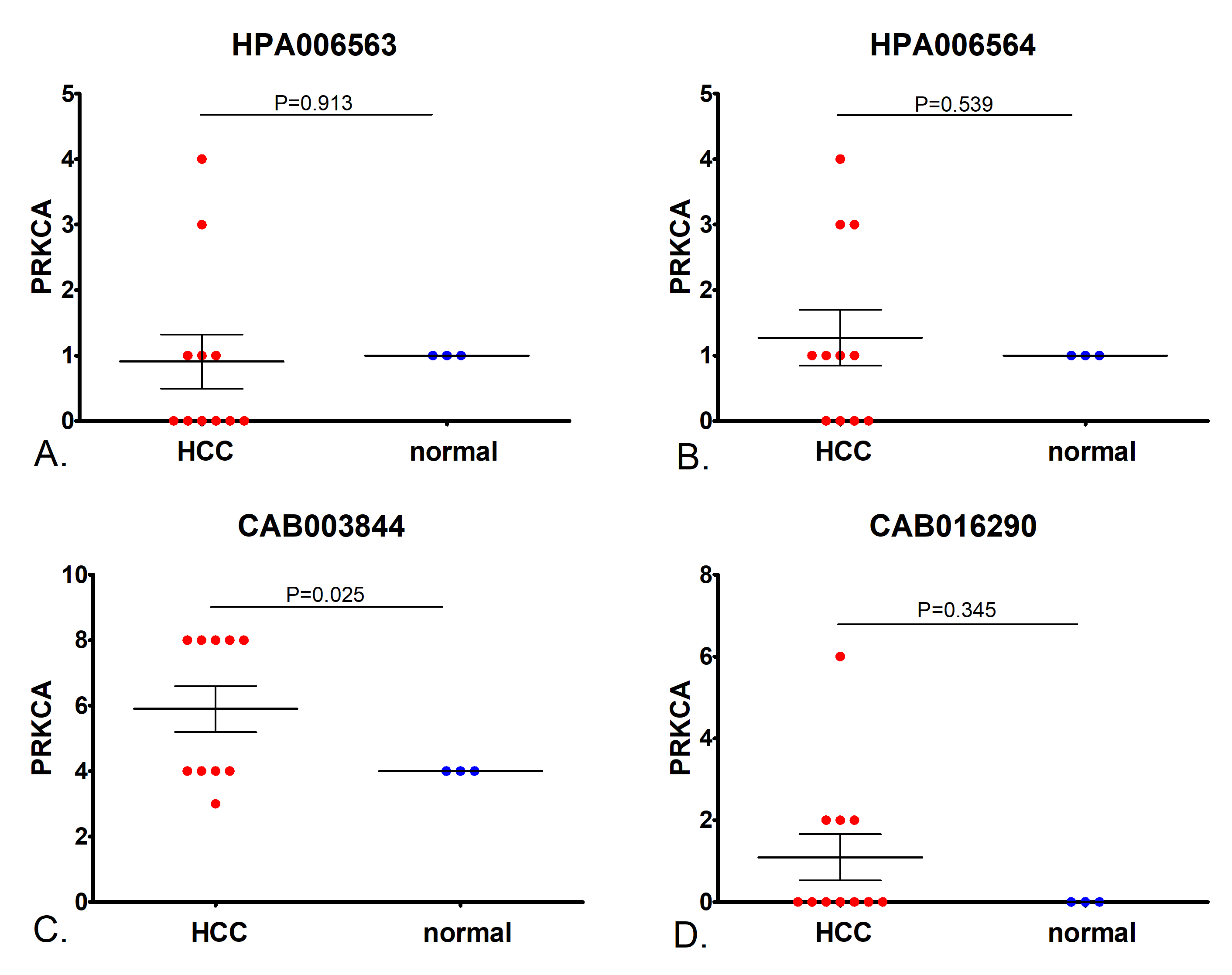


Supplementary figure 17 Immunohistochemistry results of the four antibodies of PRKCA. The t test was used to detect the different expression of PRKCA protein.

(A) antibody HPA006563. (B) antibody HPA006564. (C) antibody CAB003844. (D) CAB016290.

Supplementary Table 1

| clinical parameters | n | mean | sd | t | p |
| --- | --- | --- | --- | --- | --- |
| gender |  |  |  |  |  |
| male | 251 | 4.3389 | 1.05697 | 2.771 | 0.006** |
| female | 118 | 3.9556 | 1.31591 |  |  |
| grade |  |  |  |  |  |
| G1-G2 | 228 | 4.3856 | 1.11907 | -3.61 | <0.001*** |
| G3-G4 | 137 | 3.943 | 1.15861 |  |  |
| relapse |  |  |  |  |  |
| NO | 180 | 4.257 | 1.13268 | -0.234 | 0.815 |
| YES | 170 | 4.2286 | 1.14518 |  |  |
| M |  |  |  |  |  |
| M0 | 266 | 4.129 | 1.14717 | -0.36 | 0.719 |
| M1 | 4 | 3.9208 | 1.14273 |  |  |
| N |  |  |  |  |  |
| N0 | 253 | 4.1924 | 1.13298 | -1.092 | 0.354 |
| N1 | 4 | 2.8747 | 2.40849 |  |  |
| T |  |  |  |  |  |
| T1-T2 | 273 | 4.2335 | 1.14915 | -0.653 | 0.514 |
| T3-T4 | 94 | 4.143 | 1.18738 |  |  |
| stage |  |  |  |  |  |
| stageI-II | 256 | 4.2549 | 1.09962 | -1.409 | 0.16 |
| stageIII-IV | 90 | 4.0566 | 1.28001 |  |  |
| residual1_2 |  |  |  |  |  |
| NO | 324 | 4.2481 | 1.16139 | -1.312 | 0.19 |
| YES | 38 | 3.9866 | 1.17134 |  |  |

The correlation between clinicopathological variables and miR-125b-2-3p expression in HCC from TCGA. *P<0.05, **P<0.01, ***P<0.001

Supplementary Table 2

| clinical parameters | n | mean | sd | t | p |
| --- | --- | --- | --- | --- | --- |
| M |  |  |  |  |  |
| M0 | 6 | 1.8348 | 1.3517 | -0.825 | 0.417 |
| M1 | 20 | 4.2115 | 6.91847 |  |  |
| stage |  |  |  |  |  |
| stageI-II | 6 | 1.8348 | 1.3517 | -0.825 | 0.417 |
| stageIII-IV | 20 | 4.2115 | 6.91847 |  |  |
| thrombus |  |  |  |  |  |
| NO | 16 | 4.1866 | 7.44465 | 0.542 | 0.593 |
| YES | 10 | 2.8253 | 3.36489 |  |  |
| Nodes |  |  |  |  |  |
| multiple | 15 | 4.5892 | 7.8774 | -0.894 | 0.38 |
| single | 11 | 2.4 | 2.13273 |  |  |
| T |  |  |  |  |  |
| T1-2 | 7 | 2.7544 | 2.18945 | -0.45 | 0.657 |
| T3-4 | 19 | 3.9978 | 7.10234 |  |  |
| grade |  |  |  |  |  |
| G1-2 | 21 | 3.9031 | 6.77062 | 0.401 | 0.692 |
| G3-4 | 5 | 2.6546 | 2.31985 |  |  |
| AFP |  |  |  |  |  |
| negative | 10 | 2.126 | 1.37229 | 1.175 | 0.261 |
| positive | 13 | 4.9209 | 8.43451 |  |  |
| cirrhosis |  |  |  |  |  |
| NO | 8 | 1.7278 | 1.20503 | 1.073 | 0.294 |
| YES | 18 | 4.5231 | 7.24044 |  |  |
| gender |  |  |  |  |  |
| male | 20 | 4.1336 | 6.93185 | 0.705 | 0.487 |
| female | 6 | 2.0946 | 1.58119 |  |  |
| age |  |  |  |  |  |
| >=50 | 15 | 2.0611 | 1.98321 | -1.381 | 0.196 |
| <50 | 11 | 5.8475 | 8.93644 |  |  |

The correlation between clinicopathological variables and miR-125b-2-3p expression in HCC from in-hoouse RT-qPCR. *P<0.05, **P<0.01, ***P<0.001
